# MicroRNA-494-3p inhibits formation of fast oxidative muscle fibres by targeting E1A-binding protein p300 in human-induced pluripotent stem cells

**DOI:** 10.1038/s41598-020-80742-y

**Published:** 2021-01-13

**Authors:** Hirotaka Iwasaki, Yoshinori Ichihara, Katsutaro Morino, Mengistu Lemecha, Lucia Sugawara, Tatsuya Sawano, Junichiro Miake, Hidetoshi Sakurai, Eiichiro Nishi, Hiroshi Maegawa, Takeshi Imamura

**Affiliations:** 1 Department of Pharmacology, Shiga University of Medical Science, Otsu, Japan; 2 Division of Pharmacology, Faculty of Medicine, Tottori University, Yonago, Japan; 3 Division of Endocrinology and Metabolism, Department of Medicine, Shiga University of Medical Science, Tsukinowa, Seta, Otsu, Shiga 520-2192 Japan; 4 Center for iPS Cell Research and Application (CiRA), Kyoto University, Kyoto, Japan; 5 Department of Molecular and Cellular Biology, City of Hope, Los Angeles, USA

**Keywords:** Biophysics, Cell biology, Developmental biology, Stem cells, Endocrinology, Molecular medicine

## Abstract

MYOD-induced microRNA-494-3p expression inhibits fast oxidative myotube formation by downregulating myosin heavy chain 2 (MYH2) in human induced pluripotent stem cells (hiPSCs) during skeletal myogenesis. However, the molecular mechanisms regulating MYH2 expression via miR-494-3p remain unknown. Here, using bioinformatic analyses, we show that miR-494-3p potentially targets the transcript of the E1A-binding protein p300 at its 3′-untranslated region (UTR). Myogenesis in hiPSCs with the Tet/ON-myogenic differentiation 1 (*MYOD1*) gene (MyoD-hiPSCs) was induced by culturing them in doxycycline-supplemented differentiation medium for 7 days. p300 protein expression decreased after transient induction of miR-494-3p during myogenesis. miR-494-3p mimics decreased the levels of p300 and its downstream targets MYOD and MYH2 and myotube formation efficiency. p300 knockdown decreased myotube formation efficiency, MYH2 expression, and basal oxygen consumption rate. The binding of miR-494-3p to the wild type *p300* 3′-UTR, but not the mutated site, was confirmed using luciferase assay. Overexpression of p300 rescued the miR-494-3p mimic-induced phenotype in MyoD-hiPSCs. Moreover, miR-494-3p mimic reduced the levels of p300, MYOD, and MYH2 in skeletal muscles in mice. Thus, miR-494-3p might modulate MYH2 expression and fast oxidative myotube formation by directly regulating p300 levels during skeletal myogenesis in MyoD-hiPSCs and murine skeletal muscle tissues.

## Introduction

The skeletal muscle comprises muscle fibres with different patterns of gene expression, which lead to differences in contractile dynamics and metabolic properties. In humans, muscle fibres are classified into three types based on different myosin heavy chain (MYH) isoforms. These isoforms determine muscle-fibre function during development and adulthood^1^. MYH7 is a marker for type I slow-twitch oxidative myofibres, MYH2 is a marker for type IIa fast-twitch oxidative myofibres, and MYH1 is a marker for type IIx fast-twitch glycolytic myofibres^2,3^. Myofibre composition correlates with pathophysiology of certain disorders such as diabetes^4^, sarcopenia^5,6^, and muscular disease^7,8^. Differentiation of skeletal muscle is driven by basic helix-loop-helix transcription factors called muscle regulatory factors (MRFs) and MYOD is one of the well-characterised MRFs. MYOD can induce the differentiation of fibroblasts into myotubes^9^ and can bind to histone acetyltransferases (HATs) including p300^10^. Genetic studies in embryonic stem (ES) cells have shown that intrinsic HAT activity of p300 is specifically required for skeletal myogenesis and *MYOD* gene expression^11^. p300-dependent hyperacetylation also regulates muscle wasting^12^.

MicroRNAs (miRNAs) are small non-coding RNA molecules, typically 19- to 24-bp, that are processed by Dicer to generate their mature form^13^. miRNAs suppress multiple genes by destabilisation or reduction of translation^14^. Myogenic processes are also regulated by miRNAs^15,16^. Muscle-specific miRNAs such as miR-1, -133, -206, -208, -208b, -486, and -499 have been demonstrated to participate in physiological and pathological skeletal muscle processes such as myogenesis, regeneration, hypertrophy, and muscular dystrophy^17–23^. Among them, miR-1, -206, and -486 are induced by MYOD expression and their expression is increased during myogenesis^24,25^. Moreover, miR-208b and miR-499 control fibre type and muscle performance^21^. Recent studies also support the involvement of non-muscle-specific miRNAs in myogenesis^26–29^. We found that among these non-muscle-specific miRNAs, the expression of miR-494-3p was transiently induced by MYOD induction and reduced during myogenesis^30^. Previous studies showed that miR-494-3p expression was decreased by endurance exercise in mouse skeletal muscles^31,32^. We have also reported that miR-494-3p expression represses type IIa fast-twitch oxidative muscle fibre differentiation in a skeletal myogenesis model, using human iPS cells stably expressing the Tet/ON*-MYOD1* gene (MyoD-hiPSCs)^30^. Since miR-494-3p showed unique expression patterns and was regulated by exercise, we focused on miR-494-3p in this study. miR-494-3p regulates multiple genes such as *PTEN*^33^, *SIRT1*^34^, *c-Myc*^34^, *PDE4D*^35^, *cyclinD1*^36^, *Rab5a*^37^, *PAK1*^38^, *IGF1R*^39^, *Tfam*^31^, *Foxj3*^31^, and *Pgc1α*^40^. Although overexpression of miR-494-3p in MyoD-hiPSCs decreased the protein expression of MYH2, the underlying mechanism remains unclear, as no recognition site of miR-494-3p in the 3′-UTR of the *MYH2* gene has been found.

Based on our previous findings, we hypothesised that target proteins of miR-494-3p may be involved in the machinery regulating fibre type-specific skeletal myogenesis. Thus, in this study, we aimed to identify the direct target of miR-494-3p during fibre-type-specific skeletal myogenesis.

## Results

### MiR-494-3p downregulated protein expression of p300 during human-skeletal myogenesis in MyoD-hiPSCs

**Figure 1:**
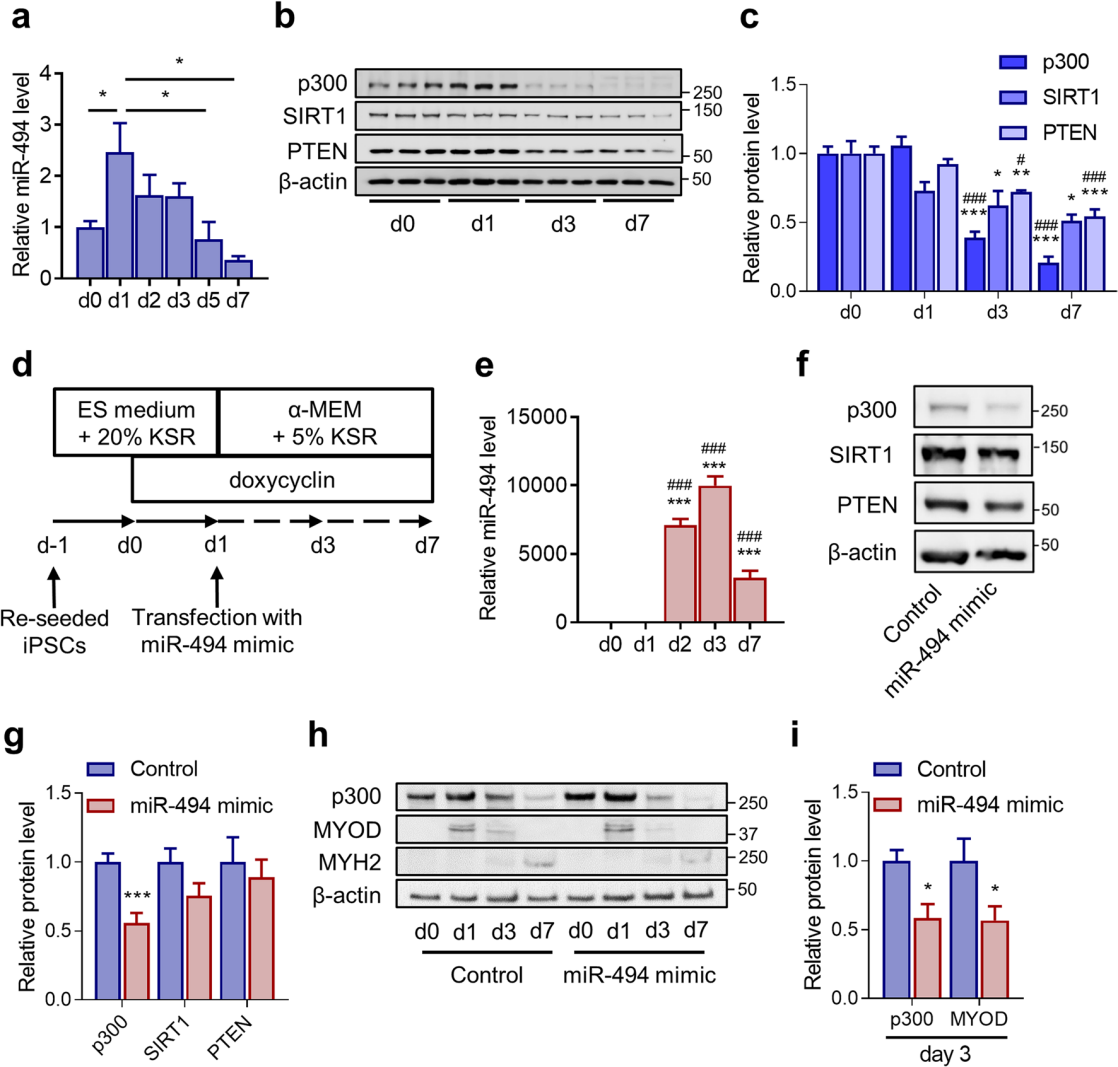
Effects of miR-494 mimic on human-skeletal myogenesis in MyoD-hiPSCs. (**a**) Time course of miR-494 expression during MyoD-hiPSC differentiation, as analysed by Taqman RT-qPCR. Data were normalised with snRNA U6. n = 4–6. *p < 0.05. (**b,c**) Time course of p300, SIRT1, and PTEN expression during MyoD-hiPSC differentiation, as analysed by immunoblotting. Expression of p300, SIRT1, and PTEN was quantified using Image J and normalised to β-actin expression. n = 3. *p < 0.05, **p < 0.01, ***p < 0.001 versus day 0. ^#^*p* < 0.05, ^###^*p* < 0.001 versus day 1. Full-length blots are presented in Supplementary Figure S4. (**d**) Schematic diagram showing transfection of MyoD-hiPSCs with miR-494 mimic at day 1. (**e**) Time course of miR-494 expression during differentiation of MyoD-hiPSCs transfected with miR-494 mimic, as analysed by Taqman RT-qPCR. Data were normalised with snRNA U6. n = 3. ***p < 0.001 versus day 0. ^###^*p* < 0.001 versus day 1. (**f,g**) MyoD-hiPSCs were transfected with miR-494 mimic or negative control duplexes (Control). Whole-cell extracts were harvested at day 7 after myogenic induction; expression of p300, SIRT1 and PTEN was evaluated using immunoblots. Data were normalised to β-actin. n = 6. ***p < 0.001 versus control. Full-length blots are presented in Supplementary Figure S5. (**h**) Time course of p300, MYOD, and MYH2 expression during differentiation of MyoD-hiPSCs transfected with miR-494 mimic or Control, as analysed by immunoblotting. Full-length blots are presented in Supplementary Figure S6. (**i**) MyoD-hiPSCs transfected with miR-494 mimic or Control were harvested at day 3 after myogenic induction; expression of p300 and MYOD was evaluated using immunoblots. Data were normalised to β-actin. n = 5. *p < 0.05 versus control.

We first searched for potential direct targets of miR-494-3p, which regulate fibre-specific skeletal myogenesis, using miRNA target prediction software. miR-494-3p expression in MyoD-hiPSCs was transiently upregulated 24 h after myogenic induction and was reduced to basal levels at day 5, and further decrease was observed until day7 (Fig. 1a). This result indicates that potential target proteins of miR-494-3p may be decreased after myogenic induction. Thus, we tested the expression patterns of some potential target proteins during MyoD-hiPSC differentiation. We found three candidates p300, SIRT1, and PTEN, whose protein expression decreased on days 3 and 7 compared with that on day 1 (Fig. 1b,c). To examine whether this reduced expression depends on miR-494-3p, we transfected a miR-494-3p mimic into MyoD-hiPSCs on day 1 to increase miR-494-3p levels during skeletal myogenesis (Fig. 1d). Increased expression of miR-494 was observed one day after transfection (day 2), which continued to increase up to 3000-fold on day 7 compared with that before transfection; the extent of increase was then reduced compared with that on day 2 or 3 (Fig. 1e). p300 expression was significantly reduced on day 7 by exogenous expression of miR-494-3p (*p* < 0.001), whereas the levels of SIRT1 and PTEN were not changed, compared with the levels in the control (Fig. 1f,g). We also found that *p300* mRNA expression was unchanged on days 3 and 7 compared with that on day 0 during MyoD-hiPSC differentiation (Supplementary Fig. S1). Since p300 is a coactivator of MYOD, we analysed the expression of MYOD and MYH2 as downstream molecules of p300 in skeletal myogenesis to clarify the mechanism of action of miR-494-3p in myogenesis. The expression of MYOD was significantly downregulated by miR-494 overexpression compared with that in the control on day 3 (*p* < 0.05) (Fig. 1h,i). The expression of MYH2 was downregulated by miR-494 overexpression compared with that in the control on day 7 (Fig. 1h). These results suggest that p300 is a possible downstream regulator of miR-494-3p in the regulation of MYOD expression during human skeletal myogenesis.

### Effects of miR-494 inhibitor on skeletal myogenesis in MyoD-hiPSCs

Next, we performed miR-494-3p inhibitor treatments to confirm the contribution of the transient increase in miR-494-3p expression to skeletal myogenesis on day 1 in hiPSCs. MyoD-hiPSCs were transfected with miR-494-3p inhibitor on day 0 before myogenic induction (Supplementary Fig. S2a). The expression of the MYH2 protein was decreased in cells transfected with the miR-494-3p mimic, whereas it was unchanged in cells transfected with the miR-494-3p inhibitor compared with that in the control (Supplementary Fig. S2b,c). There was no difference in the number of differentiated myotubes and myotube size between cells transfected with the miR-494-3p inhibitor and the control (Supplementary Fig. S2d–f).

### Effects of p300 knockdown on human-skeletal myogenesis in MyoD-hiPSCs

**Figure 2:**
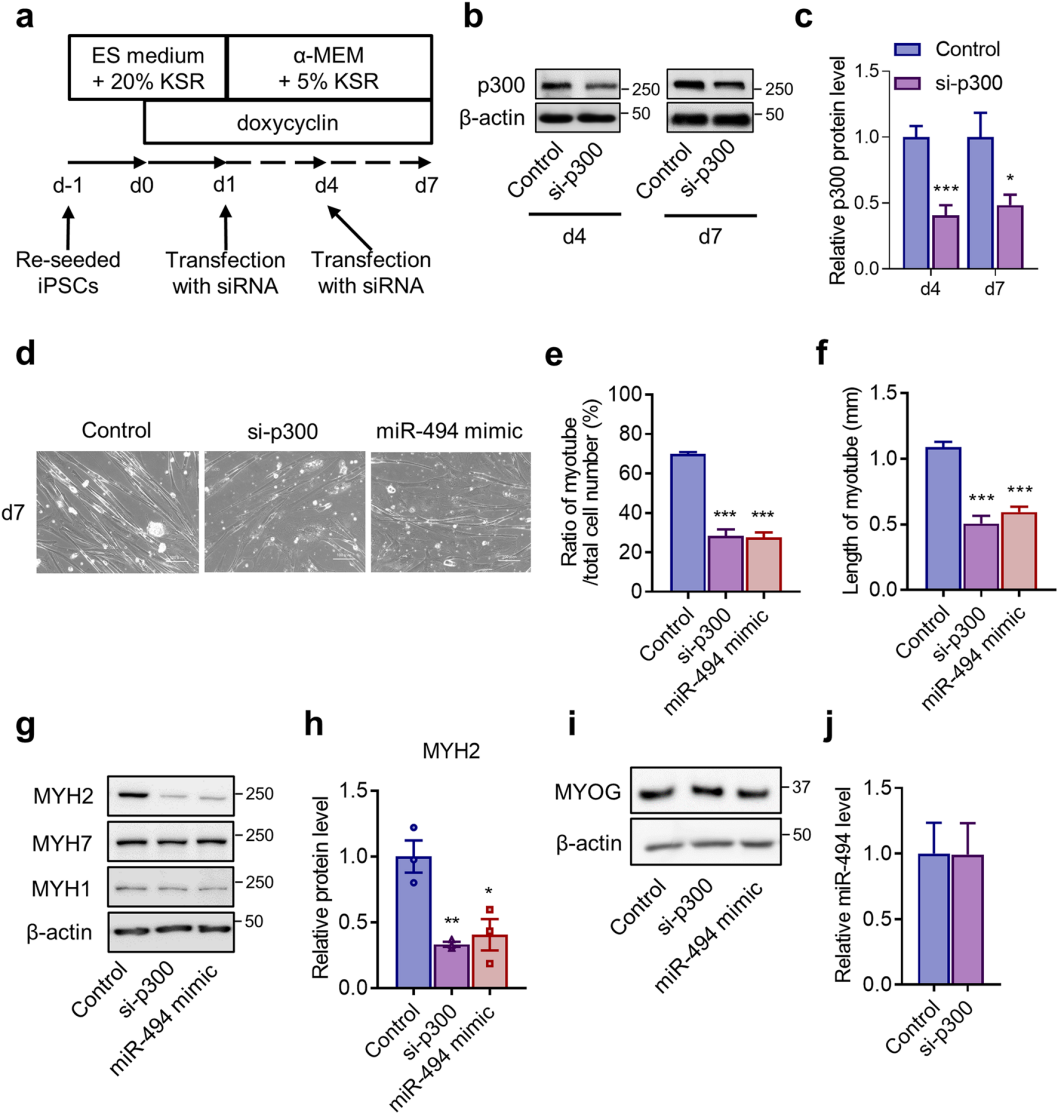
Effects of p300 knockdown on human-skeletal myogenesis in MyoD-hiPSCs. (**a**) Schematic diagram showing transfection of MyoD-hiPSCs with siRNA against p300 (si-p300) at days 1 and 4. (**b,c**) MyoD-hiPSCs were transfected with si-p300 or negative control duplexes (Control). Whole-cell extracts were harvested at day 4 and 7 after myogenic induction, and p300 expression was evaluated using immunoblotting. p300 expression was quantified using Image J and normalised to β-actin. n = 5. *p < 0.05, ***p < 0.001 versus control. Full-length blots are presented in Supplementary Figure S8. (**d**) Representative images of MyoD-hiPSCs at day 7 post-transfection with Control, si-p300, and miR-494 mimic. Scale bars: 100 µm. (**e,f**) The number (**e**) and length (**f**) of differentiated myotubes at day 7 was analysed in each microscopic field. n = 10 (**e**), n = 7 (**f**). ***p < 0.001 versus control. (**g**) Expression of myosin heavy chain 2 (MYH2), MYH7, and MYH1 in MyoD-hiPSCs transfected with Control, si-p300, or miR-494 mimic at day 7 after myogenic induction, as assessed via western blotting. Full-length blots are presented in Supplementary Figure S9. (**h**) The level of MYH2 was quantified using Image J and normalised to β-actin. n = 5. *p < 0.05, **p < 0.01 versus control. (**i**) Expression of myogenin (MYOG) in MyoD-hiPSCs transfected with Control, si-p300, or miR-494 mimic at day 7 after myogenic induction, as assessed via western blotting. Full-length blots are presented in Supplementary Figure S10. (**j**) miR-494 expression in MyoD-hiPSCs transfected with Control and si-p300 at day 7 after myogenic induction, as analysed by Taqman RT-qPCR. Data were normalised with snRNA U6. n = 3.

To confirm the contribution of p300 to skeletal myogenesis in hiPSCs, we performed siRNA-induced knockdown of p300. MyoD-hiPSCs were transfected with specific siRNAs against *p300* (si-p300) on days 1 and 4 after myogenic induction (Fig. 2a). Compared with that in control, si-p300 reduced p300 protein expression to approximately 50% on days 4 and 7 (Fig. 2b,c). This result shows that the protein expression of p300 was consistently reduced from day 4 to 7 during skeletal myogenesis. The ratio of myotube formation, assessed as the number of cells in myotubes to that of total cells, showed a 60% decrease regarding cells with si-p300 compared with the control. Moreover, compared with that in control, si-p300 reduced the length of myotubes to approximately 50% (Fig. 2d–f). This reduction followed the results of miR-494-3p overexpression (Fig. 2d–f). Myotube formation was also evaluated by fusion index, showing a 60% decrease in cells treated with si-p300 compared with myotube formation in the control, which was similar to that in cells treated with miR-494 mimic (Supplementary Fig. S3). Western blotting revealed that the level of MYH2, a specific marker for type IIa myofibres, was reduced by both si-p300 and the miR-494-3p mimic (Fig. 2g,h). The levels of MYH7, a specific marker for type I myofibres, and MYH1, a specific marker for type IIx myofibres, were unaffected by si-p300 or miR-494-3p mimic (Fig. 2g). The myogenin (MYOG) level was unchanged for both si-p300 and miR-494 mimic treatments (Fig. 2i). miR-494 expression was also unaffected by si-p300 on day7 (Fig. 2j). These results suggest that p300 is a possible downstream target of miR-494-3p during skeletal myogenesis in hiPSCs.

### Metabolic effects of p300 knockdown on skeletal myogenesis in MyoD-hiPSCs

**Figure 3:**
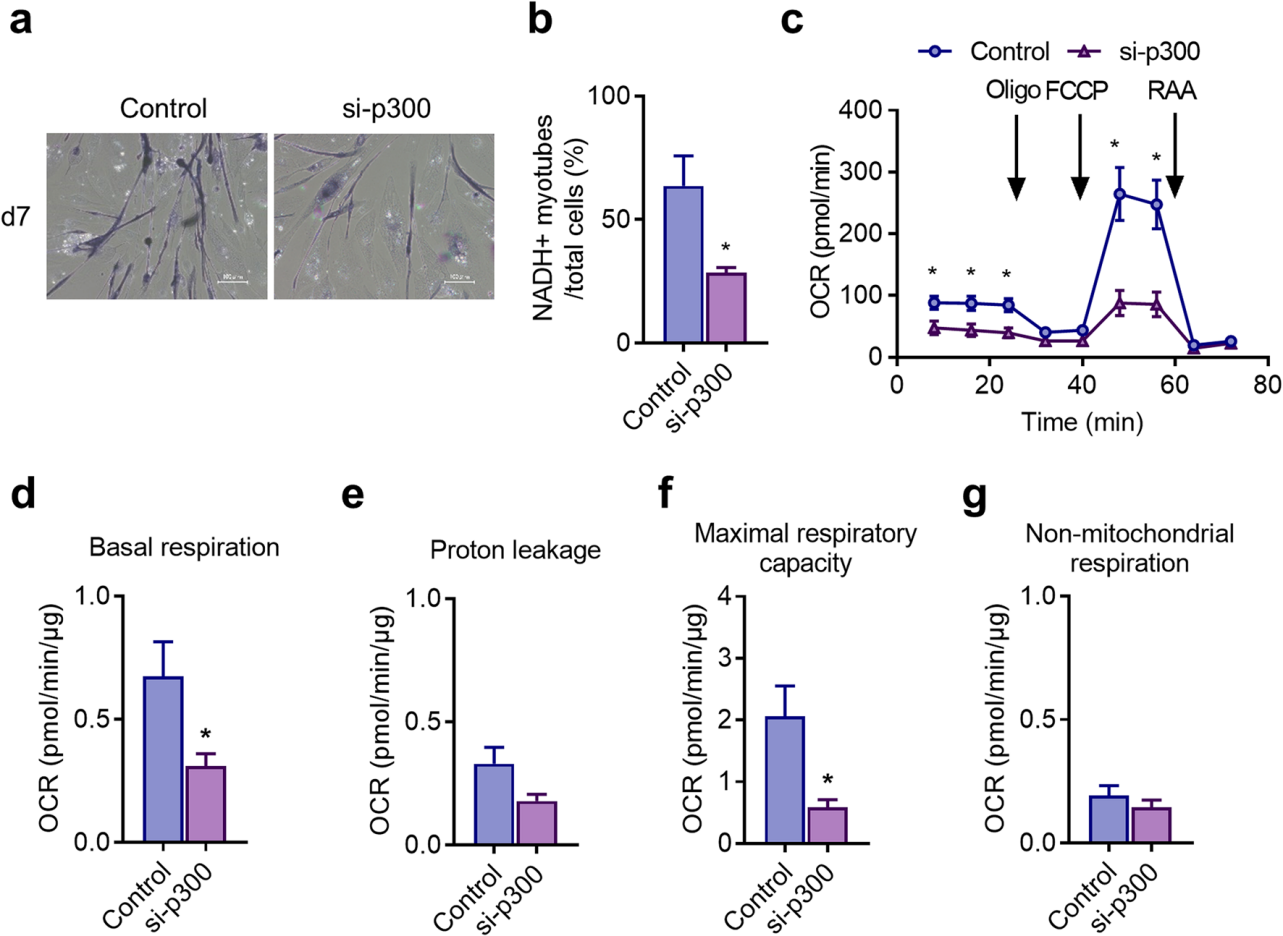
Metabolic effects of p300 knockdown on human-skeletal myogenesis in MyoD-hiPSCs. (**a**) NADH-TR staining detected myotube fibre types in MyoD-hiPSCs transfected with si-p300 or Control at day 7 after myogenic induction. Representative images are presented. Scale bars: 100 µm. (**b**) The graph shows the ratio of the number of stain-positive myotubes to total cell number counted in each microscopic field (n = 10). *p < 0.05 versus control. (**c**) MyoD-hiPSCs transfected with si-p300 or Control were evaluated using flux analyser at day 7 after myogenic induction. Levels of basal oxygen-consumption rate (OCR) were measured, and mitochondrial stress tests were performed using oligomycin (Oligo), FCCP, and rotenone-antimycin A. A representative example of five independent experiments is shown. (**d**–**g**) After the OCR measurements, cells were lysed, and protein concentration was measured to normalise OCR data obtained for basal respiration (**d**), proton leakage (**e**), maximal respiratory capacity (**f**), and non-mitochondrial respiration (**g**). n = 5. *p < 0.05 versus control.

We used NADH-TR staining to examine the oxidative activity of cells treated with si-p300 because miR-494-3p overexpression inhibits the formation of fast oxidative type IIa myotubes^30^. Compared with that in control, p300 knockdown decreased the ratio of NADH-positive cells to total cells in myotubes by 40% (Fig. 3a,b). We then examined the myotube metabolism of MyoD-hiPSCs by evaluating their oxygen consumption rate using a flux analyser. Compared with that in the control, basal respiration in cells with si-p300 decreased by 50% on day 7 after myogenic induction (Fig. 3c,d). The maximal respiratory capacity of cells with si-p300 was decreased by 70% compared with the control (Fig. 3c,f). Proton leakage and non-mitochondrial respiration were unchanged (Fig. 3c,e,g). These results indicate that mitochondrial content in the muscle cells treated with si-p300 decreased, leading to a weaker oxidative phenotype. p300 expression was essential for both formation and metabolism of highly oxidative myotubes. This follows the results of miR-494-3p overexpression shown in our previous study^30^. Namely, miR-494-3p overexpression reduces the formation of fast oxidative myotube by inhibiting p300 expression. MYH2 is a marker for a fast oxidative fibre containing more mitochondria.

### Overexpression of p300 rescued the effects of miR-494 in human-skeletal myogenesis

**Figure 4:**
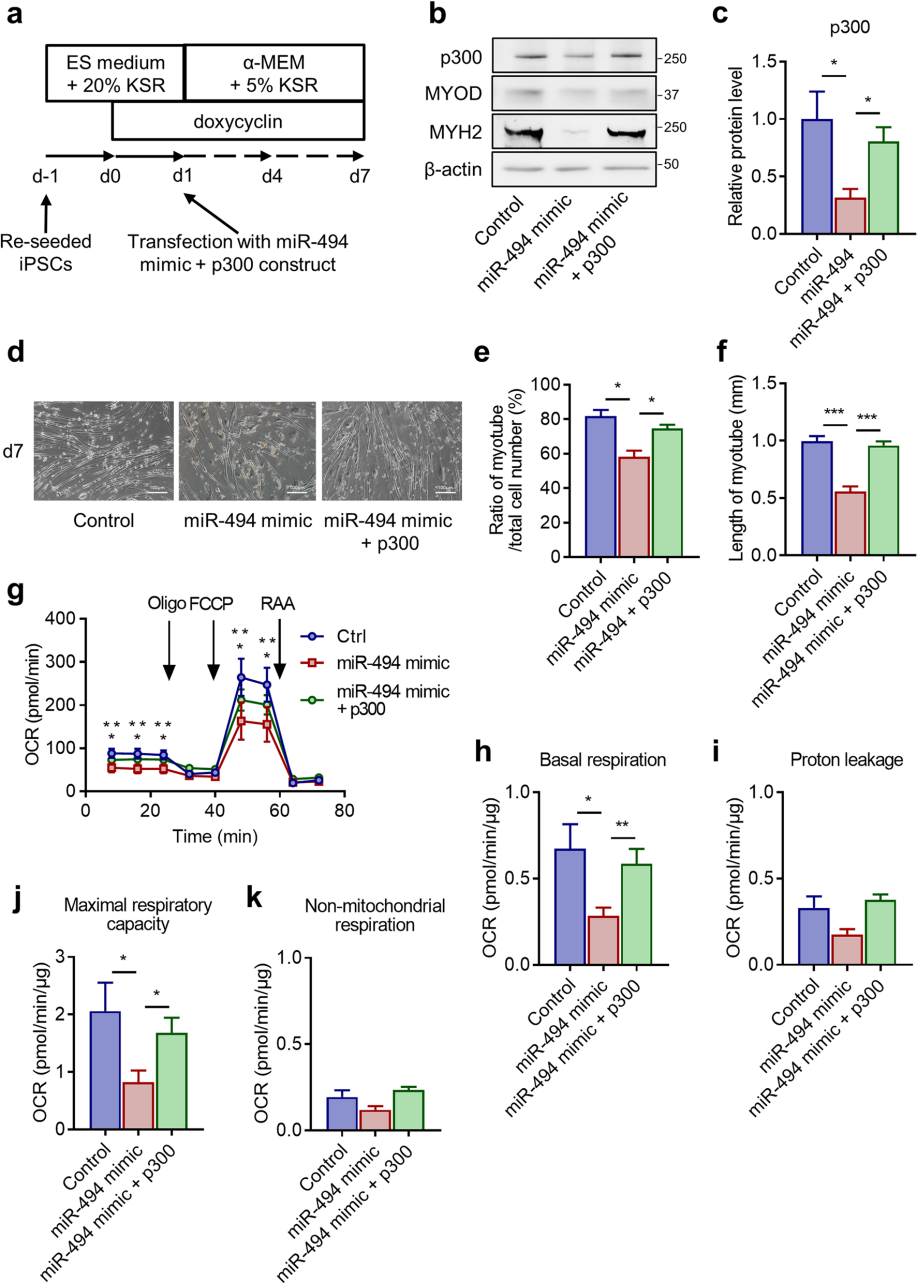
Effects of p300 overexpression on human-skeletal myogenesis in MyoD-hiPSCs transfected with miR-494 mimic. (**a**) Schematic of co-transfection of MyoD-hiPSCs with miR-494 and *p300* construct. (**b**) MyoD-hiPSCs were transfected with scrambled control and blank vector (Control), miR-494 mimic and blank vector (miR-494 mimic) or miR-494 mimic and *p300* expression vector (miR-494 mimic + p300). Whole-cell extracts were harvested at day 7 after myogenic induction; expression of p300 and MYH2 was analysed using immunoblots. Full-length blots are presented in Supplementary Figure S11. (**c**) p300 expression was quantified using Image J and normalised to β-actin. n = 5. *p < 0.05. (**d**) Representative cell images show MyoD-hiPSCs at day 7 after transfection with Control, miR-494 mimic, or miR-494 mimic + p300. Scale bars: 100 µm. (**e,f**) The number (**e**) and length (**f**) of myotubes differentiated at day 7 was counted in each microscopic field (n = 4). Data are expressed as the ratio of experimental values to control values. n = 3. *p < 0.05, ***p < 0.001. (**g**) MyoD-hiPSCs transfected with Control, miR-494 mimic, or miR-494 mimic + p300 at day 7 after myogenic induction were evaluated by a flux analyser. We then measured basal oxygen-consumption rate (OCR) and performed a mitochondrial stress test using oligomycin, FCCP, and rotenone-antimycin A. A representative example of three independent experiments is shown. Data are expressed as the mean ± SE of five wells. Bonferroni correction was performed to evaluate the association between the groups: *p < 0.05 miR-494 mimic versus control. **p < 0.01 miR-494 + p300 versus miR-494 mimic. (**h,k**) After the OCR measurements, cells were lysed, and protein concentration was measured to normalise the OCR data obtained for basal respiration (**h**), proton leakage (**i**), maximal respiratory capacity (**j**), and non-mitochondrial respiration (**k**). n = 3. *p < 0.05, **p < 0.01.

To clarify the role of p300 as a downstream target of miR-494-3p during skeletal myogenesis from hiPSCs, rescue experiments were performed using MyoD-hiPSCs transfected with both miR-494 mimic and *p300* expression vector. We performed co-transfection with the miR-494 mimic and *p300* expression vector on day 1 from myogenic induction and analysed the expression levels of MYOD and MYH2 on day 7 (Fig. 4a). Decreased expression of p300 induced by miR-494 overexpression was recovered by co-overexpression of p300 on day 7 (Fig. 4b,c). Decreased expression of MYOD and MYH2, induced by miR-494 mimic, was also rescued by co-overexpression of p300 (Fig. 4b,c). These results indicate that overexpression of p300 at least partially abolished the effects of miR-494-3p on MyoD-hiPSCs during human-skeletal myogenesis.

Cells transfected with a miR-494-3p mimic and a blank vector showed a decrease in the number of differentiated myotubes to 70% of control cells on day 7 (Fig. 4d,e). Co-overexpression of p300 and miR-494-3p mimic restored the percentage of differentiated myotubes to 90% compared with the control (Fig. 4d,e). Moreover, compared with the control, the miR-494-3p mimic and a blank vector reduced the length of myotubes to approximately 50% (Fig. 4d,f). Co-overexpression of p300 and miR-494-3p mimic restored the percentage of the length of myotubes to 95% compared with the control (Fig. 4d,f). Flux analyser showed that cells transfected with miR-494-3p mimic and a blank vector exhibited a decrease of 40% in basal respiration and maximal respiratory capacity compared with the control on day 7 (Fig. 4g,h,j). Proton leakage and non-mitochondrial respiration, however, were unchanged (Fig. 4g,i,k). Co-overexpression of p300 and miR-494-3p mimic restored the rate of basal respiration and maximal respiratory capacity to 90% compared with the control (Fig. 4g,h,j). These data show that miR-494-3p inhibited the formation of fast-twitch oxidative fibres, and this effect was rescued by overexpression of p300 in MyoD-hiPSCs, which suggest the inhibitory effect of miR-494-3p on fast-twitch oxidative fibre formation via p300.

### *p300* is a potential target of miR-494-3p

**Figure 5:**
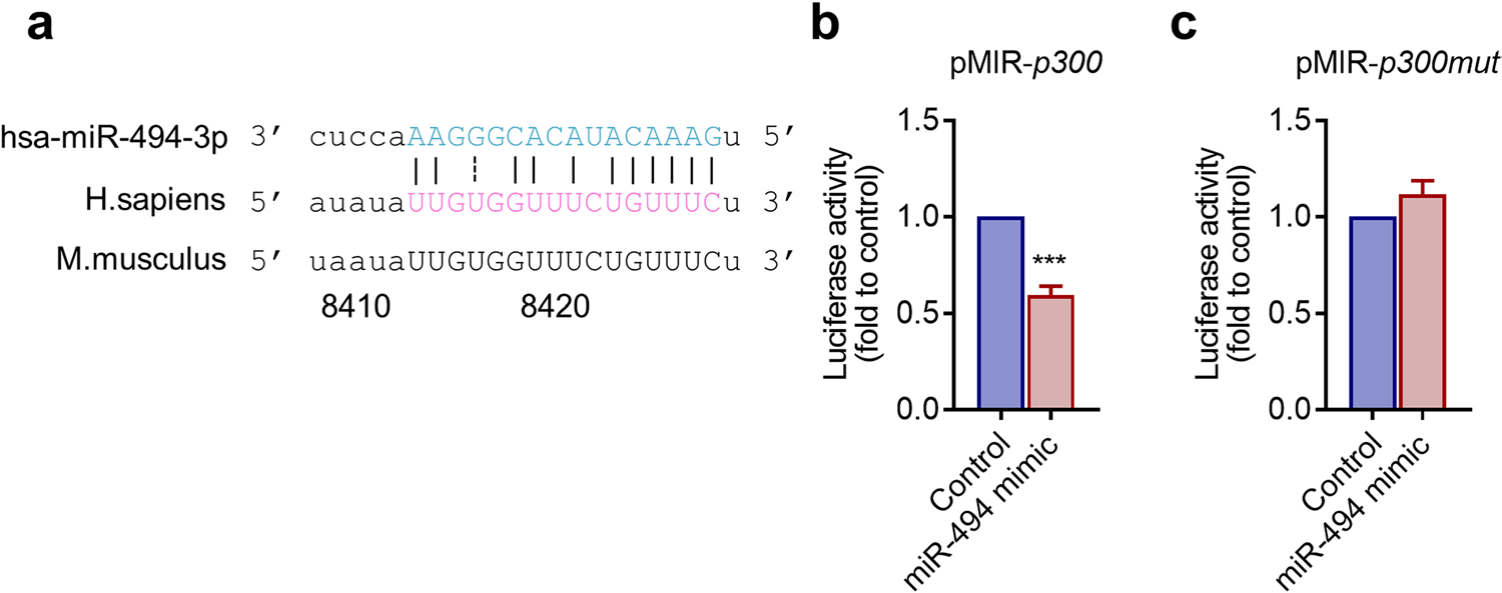
*p300* mRNA is a target of miR-494-3p. (**a**) The multiple alignments of hsa-miR-494-3p sequence, and 3′-UTR of *p300* mRNA from human and mice. The seed region of miRNA and putative target sequences in *p300* genes are shown in capital letters. (**b,c**) Luciferase reporter assays. Luciferase reporters containing the *p300* wild-type (**b**) or mutant 3′-UTR (**c**) were transfected along with miR-494-3p mimic or scrambled sequence control into MyoD-hiPSCs at day 2 after myogenic induction and analysed 2 days later. n = 5. ***p < 0.001 versus control.

Next, we explored the target of miR-494-3p in MyoD-hiPSCs. The microRNA.org prediction software identified p300 as one of the potential candidate targets of miR-494-3p. The expression of p300 was decreased in response to overexpression of miR-494-3p (Fig. 1f,g). The sequences of miR-494-3p were compared with the 3′-UTR of *p300*, and thereby a binding sequence for miR-494-3p was conserved between human and mice (Fig. 5a). We next performed a luciferase reporter assay using a construct containing the 3′-UTR region of *p300* inserted downstream of the luciferase-coding sequence. The relative luciferase activity of the wild-type *p300* 3′-UTR was significantly reduced when miR-494-3p mimics were transfected (Fig. 5b). In contrast, overexpression of miR-494-3p did not affect the expression of the luciferase reporter with the *p300* 3′-UTR containing mutations in the putative miR-494-3p-binding sequence (Fig. 5c). These data suggest a direct binding between miR-494-3p and the 3′-UTR sequence of *p300*.

### Local administration of miR-494-3p mimic inhibited p300, MYOD, and MYH2 protein expression in mouse skeletal muscle

**Figure 6:**
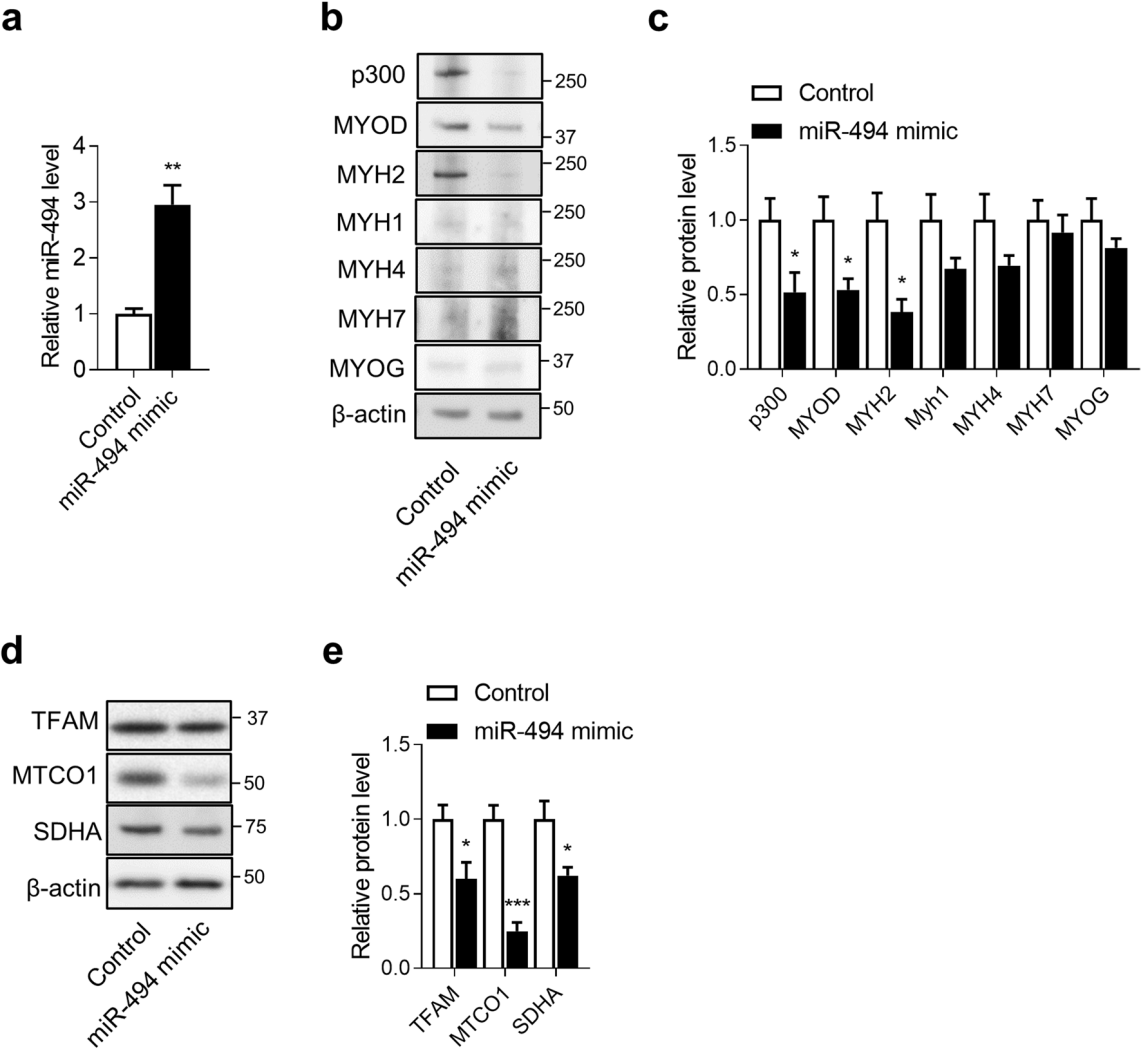
Effects of miR-494-3p mimic in TA muscle in mice. (**a**) miR-494 expression was measured by Taqman RT-qPCR in TA muscle of C57BL/6J mice injected with miRNA control mimic (Control) or miR-494-3p mimic (miR-494 mimic). Data were normalised with snRNA U6. n = 3. **p < 0.01. (**b**) p300, MYOD, MYH2, MYH1, MYH4, MYH7, and MYOG protein levels were measured by immunoblots in TA muscle of C57BL/6J mice injected with miRNA control mimic (Control) or miR-494-3p mimic (miR-494 mimic). Full-length blots are presented in Supplementary Figure S12. (**c**) The intensity of western blot bands was quantified by Image J and was normalised to β-actin. n = 6. *p < 0.05 versus control. (**d**) TFAM, MTCO1, and SDHA protein levels were measured by immunoblots in TA muscle of C57BL/6J mice injected with Control or miR-494 mimic. Full-length blots are presented in Supplementary Figure S13. (**e**) The intensity of western blot bands was quantified by Image J and was normalised to β-actin. n = 6. *p < 0.05 versus control.

We finally tested whether local overexpression of miR-494-3p could downregulate p300 expression in mice. A miR-494-3p mimic was injected in the TA muscle of one leg, and negative control mimic was injected in the contralateral leg as previously described^41–43^. miR-494-3p levels were significantly increased at about 3-times in the skeletal muscle transfected with miR-494-3p mimic (*p* < 0.01) (Fig. 6a). Injection of miR-494-3p in the TA muscle inhibited protein levels of p300 and its downstream targets, MYOD and MYH2 (Fig. 6b,c). MYH1, MYH4, MYH7, and MYOG levels were unchanged in the TA muscle treated with miR-494 mimic (Fig. 6b,c). We also examined the protein expression levels of the mitochondrial biogenesis regulators, transcription factor A, mitochondrial (TFAM), cytochrome c oxidase I (MTCO1), and mitochondrial and succinate dehydrogenase complex, subunit A (SDHA). As expected, the protein levels of them showed significant downregulation in miR-494-3p mimic-injected samples (Fig. 6d,e). These results demonstrate that miR-494-3p mediates the formation of fast oxidative type IIa myotubes in vivo.

## Discussion

In this study, we showed that the miR-494-p300-MYH2 axis regulates fast oxidative skeletal myogenesis in MyoD-hiPSCs and showed its physiological relevance in mouse muscle tissue. This study has revealed two important findings. First, the overexpression of miR-494-3p reduced p300 protein levels followed by the downregulation of MYOD and MYH2 protein expression, and attenuated the formation of fast oxidative type IIa myotubes and oxygen consumption in the differentiation of MyoD-hiPSCs. Second, the miR-494-3p mimic reduced the levels of p300 protein and its downstream targets, MYOD and MYH2, in mouse skeletal muscle. These findings demonstrate that miR-494-3p directly inhibits the expression of p300 and formation of type IIa fast-twitch oxidative myofibres in vitro and in vivo.

We showed that miR-494-3p reduced the protein expression of p300 and attenuated the formation of highly oxidative type IIa myotubes and oxygen consumption in skeletal myogenesis in MyoD-hiPSCs. We used computational miRNA target prediction algorithms and identified *p300* as one of the target genes for miR-494-3p. We confirmed the miR-494-3p-binding site in the 3′-UTR of *p300* (Fig. 5b,c), a transcriptional coregulator. This binding sequence is evolutionally conserved in mammals, including human and mouse. Previously, we showed that overexpression of miR-494-3p in MyoD-hiPSCs inhibits protein expression of MYH2 and formation of type IIa-like myotubes^30^. In this study, we found that p300 expression was reduced during myogenesis and was further reduced by the expression of miR-494-3p mimic (Fig. 1h,i). We confirmed that siRNA-induced knockdown of p300 inhibited MYH2 expression and reduced the rate of oxygen consumption and formation of mitochondria-rich myotubes (Figs. 2, 3), consistent with the effects of miR-494 overexpression. Overexpression of p300 rescued the effects of transfection with a miR-494-3p mimic (Fig. 4). These results indicate that p300 is a direct target of miR-494-3p during the formation of fast oxidative type IIa-like myotubes from human iPS cells. However, many direct target genes of miR-494-3p other than *p300* may exist, and targets of miR-494-3p may vary in each organ or cell type. A recent study in rats showed that treatment with ethanol upregulates the levels of p300 by reducing the expression of miR-494 in the amygdaloid nucleus^44^. In mouse beige adipocytes, miR-494-3p regulates mitochondrial biogenesis and thermogenesis via PGC1-α signalling^40^. In mouse skeletal muscle, miR-494-3p regulates mitochondrial biogenesis via Tfam and Forkhead box j3^31^. Other studies have shown that miR-494-3p is an oncogene and plays a central role in developing many types of solid tumours. miR-494-3p promotes hyperactivation of the PI3K/AKT pathway and progression of human hepatocellular carcinoma by targeting PTEN^33^.

As shown in Fig. 1h,i, MYOD expression was also reduced after myogenic induction during myogenesis and was further reduced by treatment with the miR-494-3p mimic on day 3. The reduction in MYOD expression could lead to many effects of the miR-494 mimic. It is important to know how miR-494 inhibits MYOD expression in the MyoD-hiPSCs system. We assume that miR-494 down regulates MYOD expression indirectly. MYOD has a positive feedback loop to stimulate its own expression. Decreased expression of p300 by miR-494-3p resulted in decreased expression of MYOD. Thus, these results suggest that the MYOD/miR-494/p300 axis is a novel pathway in regulating human skeletal myogenesis and composition of myofibres because p300 plays a role in MYOD activation^45^. As shown in Fig. 4, co-overexpression of p300 restored the expression of MYOD and MYH2, which had been reduced by the miR-494-3p mimic. Based on these results, we suggested a novel mechanism for regulating MYOD after myogenic induction. p300 regulates cell function by mediating the activity of HATs and by interacting with different transcription factors and coactivators^46–50^. p300 activates myogenic regulation and elongation factors, promoting myogenesis via its downstream effectors, MYOD and myogenic factor 5 (MYF5)^46^. Studies have shown that p300 is critical for induction of MYOD and for determination of myogenic cell fate in vivo^51^. In Tet on MyoD-hiPSCs system, exogenous MYOD was induced during Tet treatment, but protein expression of MYOD was downregulated after myogenic induction during myogenesis (Fig. 1h,i). In this study, MYOD transiently induced the expression of miR-494-3p, which downregulates p300 protein expression. p300 expression in hiPS cells was high in basal and decreased by MYOD after myogenic induction. These results suggest that p300 protein expression in hiPS cells was decreased by miR-494-3p induced by MYOD after myogenic induction. So, we assume that MYOD induced miR-494-3p has a novel negative feedback loop which decreases MYOD expression itself and downregulates downstream of MYOD regulated genes. On the other hand, p300 knockdown did not change the miR-494 expression on day 7 (Fig. 2j). Therefore, we believe p300 does not affect the expression of miR-494-3p, at least in our experimental condition.

This study has some debatable issues. First is the difference between MyoD-hiPSCs and human primary myotubes. The largest difference between the two cell types lies in the expression of MYF5 and MYH2. A previous report showed that MyoD-hiPSCs showed high similarity with primary myotubes except for MYF5 expression^52^. Moreover, another report has shown that primary myotubes express sarcomere isoforms such as MYH3, MYH7, MYH8, and MYH13, but not adult fast skeletal muscle isoforms (MYH1, MYH2, and MYH4)^53^. This discrepancy could be a strength of MyoD-hiPSCs.

The second issue is the time lag between p300 and miR-494 expression. As shown in Fig. 1a, miR-494-3p expression increased from day 0 to day 1 and then decreased. However, the protein expression of p300 was stable on days 0 and 1 and decreased only on day 3 (Fig. 1b). It is unclear how these endogenous levels are directly correlated. We speculate that the increase in miR-494 expression on day 1 inhibited p300 with some time lag. Previous reports have shown similar time lags between miRNA-induced inhibition of translation and the target protein content^54–57^. The half-life of p300 is reported to be approximately 11–14 h in several cells lines^58–61^. We also found that p300 mRNA expression was unchanged on day 3 compared with that on day 0 during MyoD-hiPSC differentiation (Supplementary Fig. S1). This result suggests post-transcriptional regulation of p300 during MyoD-hiPSC differentiation.

Third, the MYH2 protein expression and the number of differentiated myotubes were unchanged in cells transfected on day 0 with miR-494-3p inhibitor (Supplementary Fig. S2). The reason underlying these results remains unknown. In our previous study, experiments conducted with miR-494 inhibitor showed there was no difference in the number of differentiated myotubes and oxygen consumption between cells transfected with the miR-494 inhibitor and those transfected with the scrambled control on day 7^30^. These results suggest that the efficacy of miR-494 inhibitor may be too weak. Alternatively, there might be a mechanism compensating for the effects of miR-494 knockdown, potentially by upregulating other miRNAs.

Human skeletal muscle fibre composition is affected by diseases such as type 2 diabetes and sarcopenia. Thus, the fourth issue is how miR-494-3p expression is affected by type 2 diabetes and sarcopenia. To date, no study has shown the significance of miR-494 in the skeletal muscle in type 2 diabetes and sarcopenia. However, a previous study showed that miR-494 expression was upregulated by tumour necrosis factor-α and desensitised the insulin effect in C2C12 muscle cells^62^. In another study, miR-494-3p expression induced by compressive force inhibited proliferation in MC3T3-E1 cells^63^. Furthermore, miR-494-3p has been linked to insulin-resistance and type 2 diabetes^64,65^.

We found that the miR-494-3p mimic reduced the levels of p300 protein and its downstream targets, MYOD and MYH2, in murine skeletal muscle. Moreover, the levels of key mitochondrial biogenesis regulators, such as TFAM, MTCO1, and SDHA protein levels, was also reduced with miR-494-3p mimic injection in TA. Our results suggest that miR-494-3p mediates fast oxidative myotube formation and mitochondrial biogenesis in vivo. These findings corroborate those in our previous report, in which acute exercise reduced the expression of miR-494-3p in skeletal muscle in mice^31^. Other reports also showed reduced miR-494-3p expression in muscles after exercise^32,66^. The mechanisms of muscle fibre type conversion against exercise may be partially mediated by miR-494-3p, like the mechanism we observed in this study. To confirm the impact of miR-494-3p on muscle fibre type conversion against exercise, further investigations are necessary. To examine the function of miR-494-3p more clearly in skeletal muscles, we have initiated the construction of miR-494 flox mice using the CRISPR-Cas9 system. Further experiments in miR-494 knockout mice may help elucidate the role of miR-494 in skeletal muscle in vivo.

In summary, our findings proved that miR-494-3p inhibited the formation of fast oxidative myotube by directly targeting p300 in skeletal myogenesis in human iPS cells and probably in vivo as well.

## Methods

### Materials

The primary antibodies used in this study were as follows: Mouse anti-MYOD (sc-377460), anti-myosin heavy chain 7 (MYH7; sc-53089), anti-β-actin (sc-47778), monoclonal antibodies (mAbs), rabbit anti-p300 (sc-584), anti-SIRT1 (H-300), and anti-MYH2 (sc-53095) polyclonal antibodies (pAbs) were purchased from Santa Cruz Biotechnology (Dallas, TX, USA). Rabbit anti-MYH1 (25182-1-AP) and anti-MYH4 (20140-1-AP) pAb were purchased from Proteintech (Rosemont, IL, USA). Rabbit anti-PTEN (#9559) mAb was purchased from Cell Signalling Technology (Danvers, MA, USA). Mouse anti-SDHA (ab14715), rabbit anti-TFAM (ab131607) pAb, and mouse anti-MTCO1 (ab14705) mAb were purchased from Abcam (Cambridge, UK). Mouse anti-MYOG (556358) mAb was purchased from BD Pharmingen (San Diego, CA, USA). Mouse anti-human myosin heavy chain mAb was purchased from R&D systems (Minneapolis, MN, USA). Goat anti-mouse IgG-HRP (sc-2005) and goat anti-rabbit IgG-HRP (sc-2004) secondary antibodies were purchased from Santa Cruz Biotechnology (Dallas, TX, USA). All other reagents were purchased from Wako Chemicals (Kyoto, Japan).

### In-silico analysis

To analyse the target sequences of miR-494-3p, we used computational target prediction programmes (microRNA.org^67^ and TargetScanHuman^68^). We screened the target genes of miR-494-3p with evidence for target interaction, as predicted by microRNA.org (mirSVR score < − 0.5) and TargetScanHuman (cumulative weighted context++ score < − 0.2). We used these thresholds to obtain a high-confidence list of candidate targets of miR-494-3p. Next, we selected genes controlling fibre type-specific skeletal myogenesis via a literature search using the MeSH terms “Fibre-type”, “Skeletal muscle”, and “skeletal myogenesis”.

### Mice

This study was approved by the Animal Care and Use Committee of the Shiga University of Medical Science (Otsu, Japan). All animal experiments were carried out in accordance with the Guidelines for the Husbandry and Management of Laboratory Animals of the Research Center for Animal Life Science at Shiga University of Medical Science. Male C57BL/6J mice (8-week-old) were obtained from Charles River Japan (Yokohama, Japan) and maintained on a chow diet with ad libitum access to water. Mice were maintained in environmentally controlled rooms.

### In vivo transfection of miRNA mimic

Injection of miR-494-3p mimics into the TA muscles of C57BL/6J mice was performed according to manufacturer’s instruction with minor modifications. In brief, fifty microliters of 250 μM miR-494-3p mimic (MC12409, Thermo Fisher Scientific, Waltham, MA, USA) were mixed with 333 μL of Invivofectamine 2.0 reagent (Thermo Fisher Scientific, Waltham, MA, USA) and the mixture incubated for 30 min at room temperature. As a control, fifty microliters of 250 μM negative control (4464059, Thermo Fisher Scientific, Waltham, MA, USA) were used. 15 mL of 5% glucose was added to the mixture and concentrated using Amicon Ultracel-100 (Merck, Darmstadt, Germany) to 300 μL. Finally, 50 μL of miRNA complex (2.5 mg/kg) was injected into the TA muscle of one leg, and the negative control complex was injected into the contralateral leg. These procedure were repeated for 6 days and sacrificed at day 7 of injecting to harvest muscle samples.

### Human-skeletal myogenesis in MyoD-hiPS cell line

The model of human-skeletal myogenesis was established as described previously^52^. Briefly, human iPS cells [clone: 201B7, provided by CiRA (Kyoto University, Japan)] were stably transfected with a PB-TAC-ERN vector encoding myogenic differentiation 1 gene (Tet/ON-*MYOD1*)-IRES-mCherry cDNA. Then, the appropriate cell clones (MyoD-hiPSCs) were chosen via G418 selection. For myogenic differentiation, MyoD-hiPSCs were seeded onto Matrigel (BD Biosciences, San Jose, CA, USA) or Synthemax II-SC Substrate (Corning, Corning, NY, USA)-coated dishes without feeder cells. Twenty-four hours after seeding (day 0), doxycycline (1 mg/mL) was added to the ES cell medium. Twenty-four hours after induction via doxycycline (day 1), culture medium was replaced with differentiation medium composed of alpha Minimal Essential Medium (aMEM; Nacalai Tesque, Kyoto, Japan) supplemented with 5% Knockout Serum Replacement (KSR; Thermo Fisher Scientific, Waltham, MA, USA) and doxycycline (1 mg/mL), as described previously^52^. Two MyoD-hiPSCs clones (#2 and #11) were used to confirm reproducibility in most experiments performed in this study.

### Cell culture and transfection

MyoD-hiPSCs were cultured and maintained on inactive SNL feeder cells (DS Pharma Biomedical, Osaka, Japan), as described previously^52^, in primate ES cell medium (Nacalai Tesque, Kyoto, Japan) supplemented with recombinant human basic fibroblast growth factor at a concentration of 4 ng/mL (Wako Chemicals, Wako, Japan).

MyoD-hiPSCs were transfected with miR-494-3p mimic (MC12409, Thermo Fisher Scientific, Waltham, MA, USA), miR-494-3p inhibitor (MH12409, Thermo Fisher Scientific, Waltham, MA, USA), or scrambled control (AM17110, Thermo Fisher Scientific, Waltham, MA, USA) using lipofection reagent RNAiMAX (Thermo Fisher Scientific, Waltham, MA, USA) according to the manufacturer’s instructions. For overexpression of miR-494-3p, MyoD-hiPSCs were transfected with 5 nM of miR-494-3p mimic on day 1 after induction with doxycycline, and assayed on day 7 or on the day following myogenic induction. For knockdown of miR-494-3p, MyoD-hiPSCs were transfected with 5 nM of miR-494-3p inhibitor on day 0 after induction with doxycycline and assayed on day 7 after myogenic induction.

To knock down p300, MyoD-hiPSCs were transfected with 10 nM of a specific siRNA on days 1 and 4 after myogenic induction, using RNAiMAX (Thermo Fisher Scientific, Waltham, MA, USA) and then assayed on day 7. Reproducibility of all results was confirmed using two siRNAs against *p300* (s4696 and s4697; Thermo Fisher Scientific, Waltham, MA, USA).

For overexpression of p300 using a 1245 pCMVb *p300* expression vector (Addgene, Cambridge, MA, USA), MyoD-hiPS cells were co-transfected with 5 nM of miR-494 mimic and 30 nM of *p300* construct on day 1 after myogenic induction using Lipofectamine 3000 (Thermo Fisher Scientific, Waltham, MA, USA), and then assayed on day 7. Control cells were transfected with scrambled control and pCMVb blank vector using the same protocol as described above.

### Luciferase reporter assay

Luciferase reporter assay was performed as previously described^31^. In brief, dual reporter expression clones of human wild-type *p300* 3′-UTR in pEZX-MT06 vector were obtained from Genecopoeia (Rockville, MD, USA). The mutant *p300* 3′-UTR reporter was created by mutating the seed regions of the predicted hsa-miR-494-3p site (from UUGUGGUUUCUGUUUC to UUGUGGUUUCUCAAAG). The 3′-UTR sequences were inserted downstream of a firefly luciferase reporter gene driven by an SV40 enhancer. The Renilla luciferase reporter was an internal control for transfection efficiency. The plasmids were transfected into human iPS cells on day 2 after myogenic induction, using polyethyleneimine (PEI) “Max” (Polysciences, Warrington, PA, USA) according to the manufacturer’s instructions. Dual luciferase activity was measured using the Dual-Glo Luciferase Assay System (Promega, Madison, WA, USA) according to the manufacturer’s instructions on day 4.

### Nucleic acid and protein quantification

Total RNA and cellular protein were isolated using a miRvana PARIS kit (Thermo Fisher Scientific, Waltham, MA, USA) according to manufacturer’s instructions. Protein expression was examined via western blotting as described previously^52^. cDNA was prepared using PrimeScript 2 first-strand cDNA synthesis kit (Takara Bio, Otsu, Japan). RT-qPCR was preformed using SYBR Green PCR master mix (Thermo Fisher Scientific, Waltham, MA, USA) according to manufacturer's instructions. All quantitative data were normalised against expression levels of β-actin. miR-494-3p expression was analysed by RT-qPCR using a TaqMan miRNA assay kit (002365; Thermo Fisher Scientific, Waltham, MA, USA), according to the manufacturer's instructions. U6 small RNA was used as the internal control for miR quantification (001973; Thermo Fisher Scientific, Waltham, MA, USA). Primer sets used in this study are listed in Supplementary Table S1.

### Analysis of cellular metabolism

Metabolic activity in skeletal myotubes was measured as cellular oxygen consumption via XF-24 flux analyser (Seahorse Bioscience, North Billerica, MA, USA), as described previously^30^. Cells were seeded at a density of 50,000 cells per well and cultured overnight in full growth medium. After overnight attachment, the medium was washed and replaced with XF assay medium. Levels of basal OCR were measured, followed by a mitochondrial stress test. Each value was corrected regarding protein concentration, and all measurements were repeated at least three times.

### Live cell staining

Live cell staining was performed as previously described^30^. In brief, for nicotinamide adenine dinucleotide-tetrazolium reductase (NADH-TR) staining, cells were incubated with NADH-TR solution (56 mM Tris-base; 0.16 M Tris–HCl, pH 7.4; 1.2 mM Nitro blue tetrazolium; and 1.0 mM NADH) for 30 min at 37 °C and then washed with phosphate buffered saline. Images were captured using a BZ-9000 (Keyence, Osaka, Japan) and the positive cells were counted manually. Over three images per dish were counted, and the average values were used to calculate the positive cell ratio in the experiment. We have shown the means of 10 independent experiments in graphs.

### Assessment of myotube length

Myotube length was determined on day 7. Images were captured using a BZ-9000 (Keyence, Osaka, Japan). The myotube length was determined by measuring the average length of long multinucleate fibres using the Image J software. For estimating the mean value of myotube length, the five largest myotubes in five fields for each of the three wells per sample were measured.

### Quantitative analysis of the fusion index

The fusion index was analysed as described previously^69^. On day 7 after inducing differentiation, myotubes were immunostained with anti-myosin heavy chain and DAPI. Images were captured using a BZ-9000 (Keyence, Osaka, Japan) and the fusion index was calculated as the ratio of the nuclei number in myotubes (cells having over two nuclei) to the total nuclei number from five random fields for each experiment. We show the means of four independent experiments in graphs.

### Statistical analysis

All data were expressed as mean ± SEM. We performed statistical analysis by using GraphPad Prism7 software (San Diego, CA, USA). The significance of differences between two groups were evaluated by Student’s *t*-test. One-way ANOVA and Bonferroni’s multiple comparisons test were used to determine differences between the multiple groups. *p* < 0.05 was considered significant.

### Conference Presentation

A part of the current study was presented at the 18th World Congress of Basic and Clinical Pharmacology in Kyoto, Japan from July 1st to 6th, 2018.

## Supplementary Information

Supplementary Information.

## Data Availability

No datasets were generated or analysed during the current study.
