# Supplementary material for: MicroRNA-494-3p inhibits formation of fast oxidative muscle fibres by targeting E1A-binding protein p300 in human-induced pluripotent stem cells
Source: Sci Rep. 2021 Jan 13;11:1161. doi: 10.1038/s41598-020-80742-y (PMC7806978; doi:10.1038/s41598-020-80742-y)
Supplement: Supplementary file 1 — Supplementary Information. [file 41598_2020_80742_MOESM1_ESM.pdf]

# Online Supplement

## MicroRNA-494-3p inhibits formation of fast oxidative muscle fibres by targeting E1A-binding protein p300 in human-induced pluripotent stem cells

Hirotaka Iwasaki[a], Yoshinori Ichihara[b], Katsutaro Morino[c*], Mengistu Lemecha[c#], Lucia Sugawara[c], Tatsuya Sawano[b], Junichiro Miake[b], Hidetoshi Sakurai[d], Eiichiro Nishi[a], Hiroshi Maegawa[c], Takeshi Imamura[b]

[a]Department of Pharmacology, Shiga University of Medical Science, Japan
[b]Division of Pharmacology, Faculty of Medicine, Tottori University, Japan
[c]Division of Endocrinology and Metabolism, Department of Medicine, Shiga University of Medical Science, Japan
[d]Center for iPS Cell Research and Application (CiRA), Kyoto University, Japan

*Address correspondence to:
Katsutaro Morino, M.D., Ph.D.
Division of Endocrinology and Metabolism, Department of Medicine, Shiga University of Medical Science, Japan
Tsukinowa, Seta, Otsu-city, Shiga, 520-2192, Japan
Phone: +81-77-548-2222
Fax: +81-77-543-3858
E-mail: morino@belle.shiga-med.ac.jp

[#]Current affiliation: Department of Molecular and Cellular Biology, City of Hope, Los Angeles, USA

## Supplementary Figure S1.

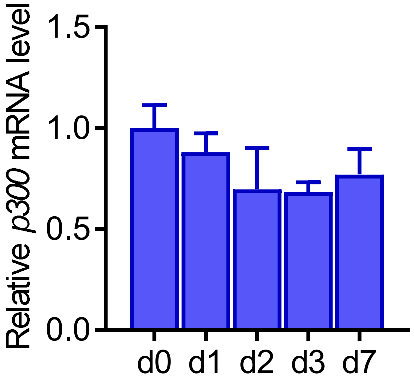


**Supplementary Figure S1. Time course of *p300* expression during MyoD-hiPSCs differentiation.** Time course of *p300* as analysed by RT-qPCR. Data were normalised with *β-actin*. n = 3.

# Supplementary Figure S2.

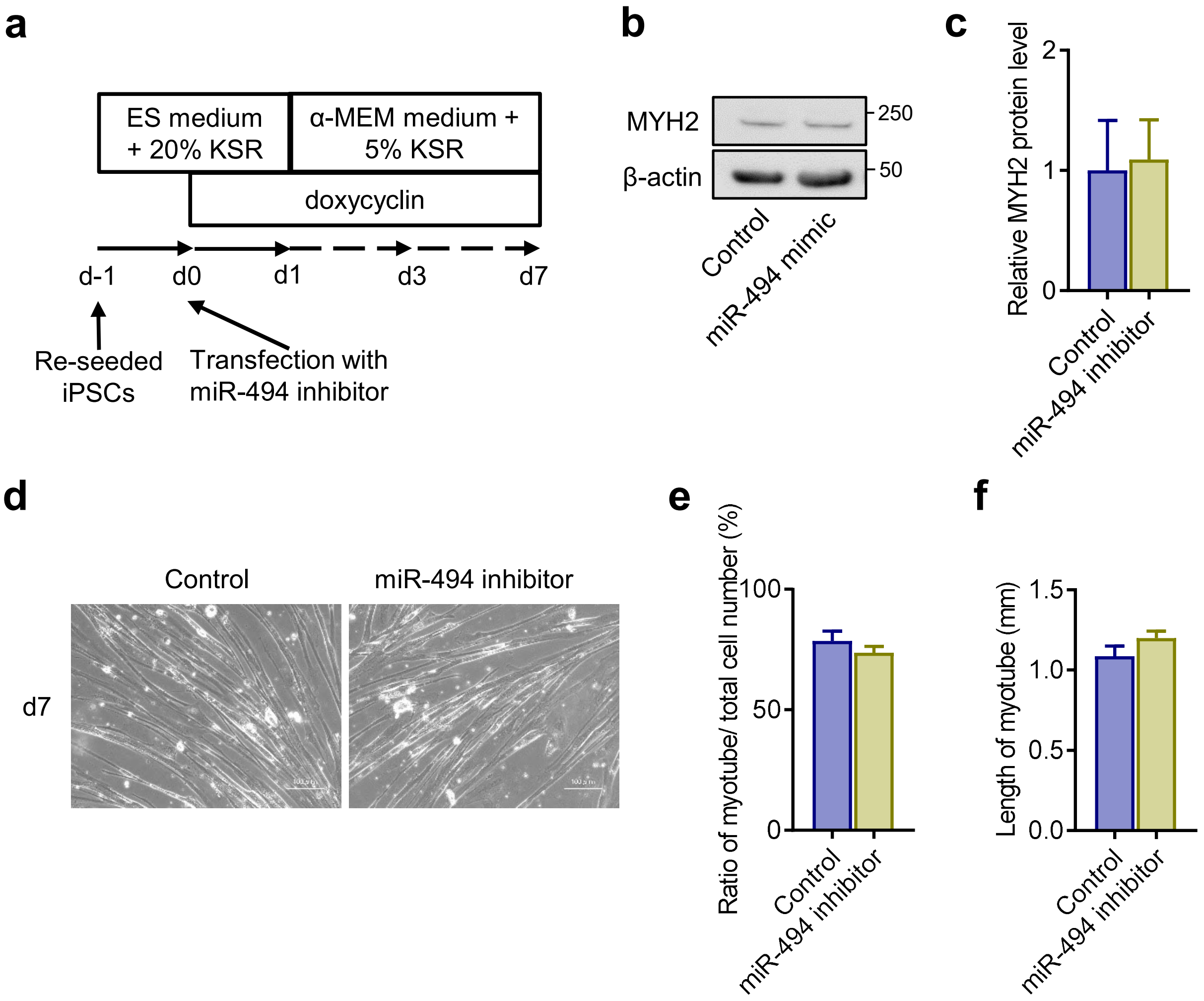


**Supplementary Figure S2. Effects of day0 transfection with miR-494-3p inhibitor on human-skeletal myogenesis in MyoD-hiPSCs. a**) Schematic diagram showing transfection of MyoD-hiPSCs with miR-494-3p inhibitor at day0. **b,c**) MyoD-hiPSCs were transfected with miR-494-3p inhibitor or negative control duplexes (Control). Whole-cell extracts were harvested at day 7 after myogenic induction, and MYH2 expression was evaluated using immunoblotting. Expression of MYH2 was quantified using Image J and normalised to β-actin expression. n = 6. Full-length blots are presented in Supplementary Figure 5. **d**) Representative images of MyoD-hiPSCs at day 7 post-transfection with Control and miR-494 inhibitor. **e,f**) The number (**e**) and length (**f**) of differentiated myotubes at day 7 was analysed in each microscopic field. n=3.

# Supplementary Figure S3.

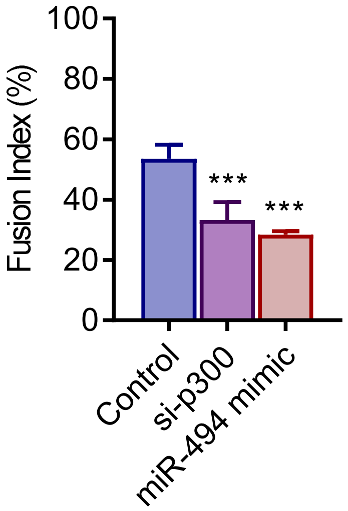


**Supplementary Figure S3. Fusion index in MyoD-hiPSCs transfected with siRNA against *p300* or miR-494 mimic.** Fusion index was calculated at day 7 as the ratio of the number of nuclei in the myotubes to the total number of nuclei in 5 random fields from the four independent experiments, ***$p < 0.001$.

**Supplementary Figure S4.**

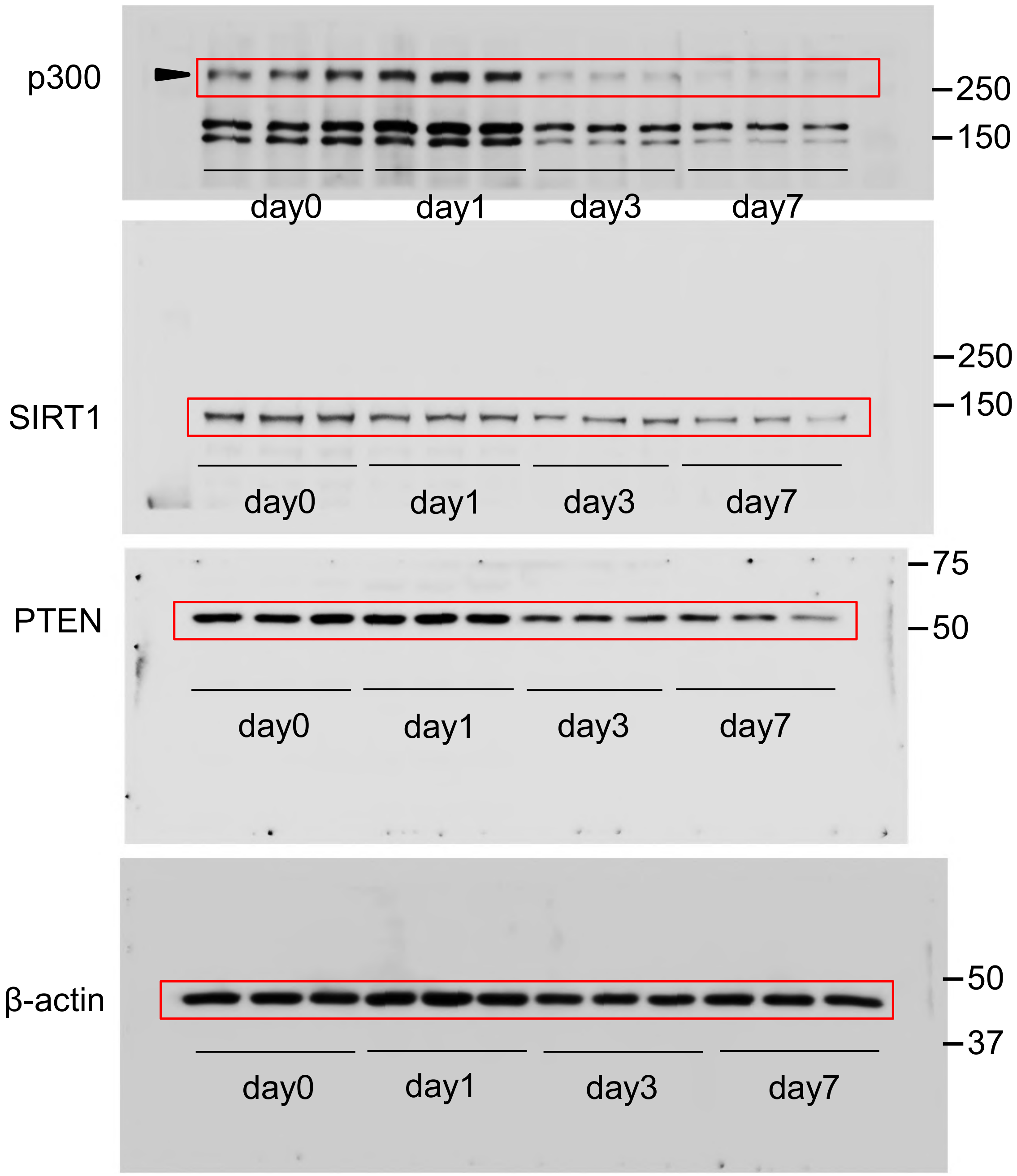


Original western blots for the images shown in Figure 1b. The regions of original blots used in main figures were encircled with red boxes.

**Supplementary Figure S5.**

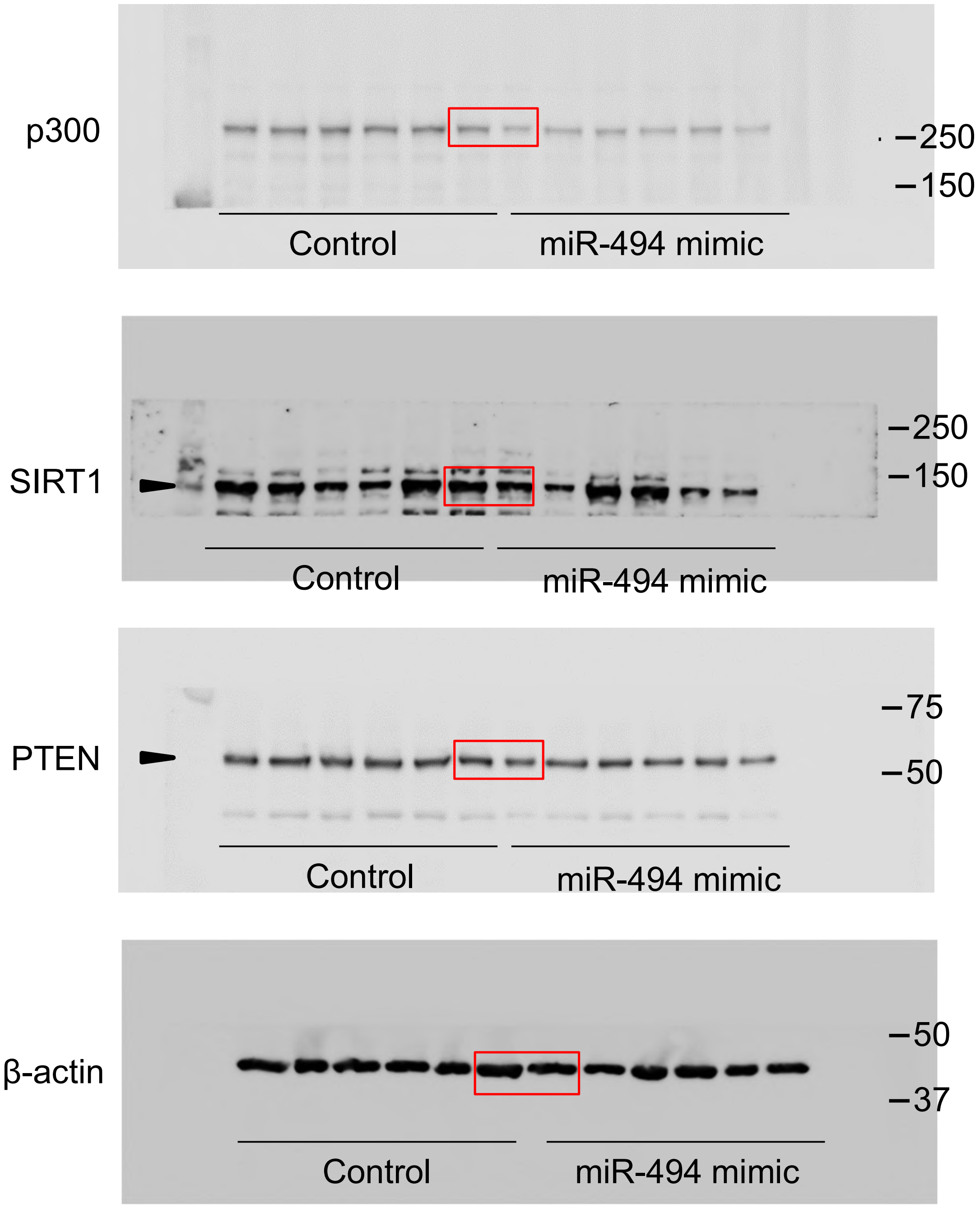


Original western blots for the images shown in Figure 1f. The regions of original blots used in main figures were encircled with red boxes.

**Supplementary Figure S6 (to be continued).**

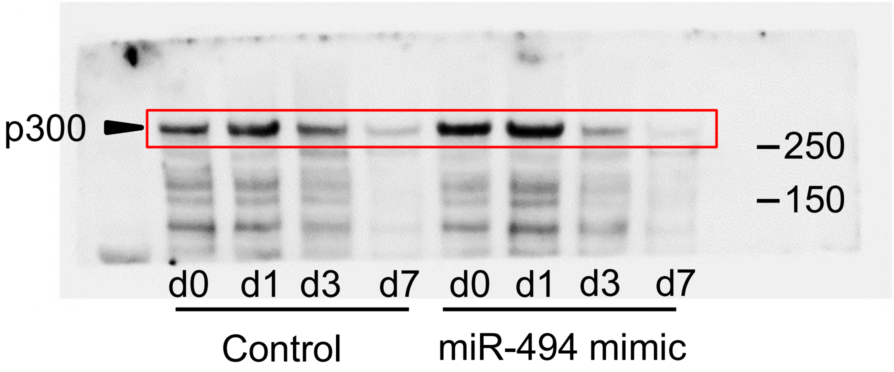


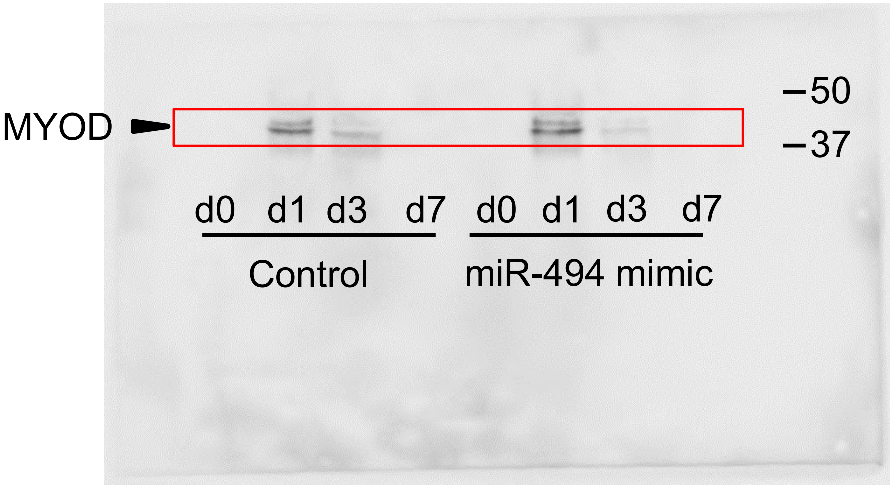


Original western blots for the images shown in Figure 1h. The regions of original blots used in main figures were encircled with red boxes.

## Supplementary Figure S6.

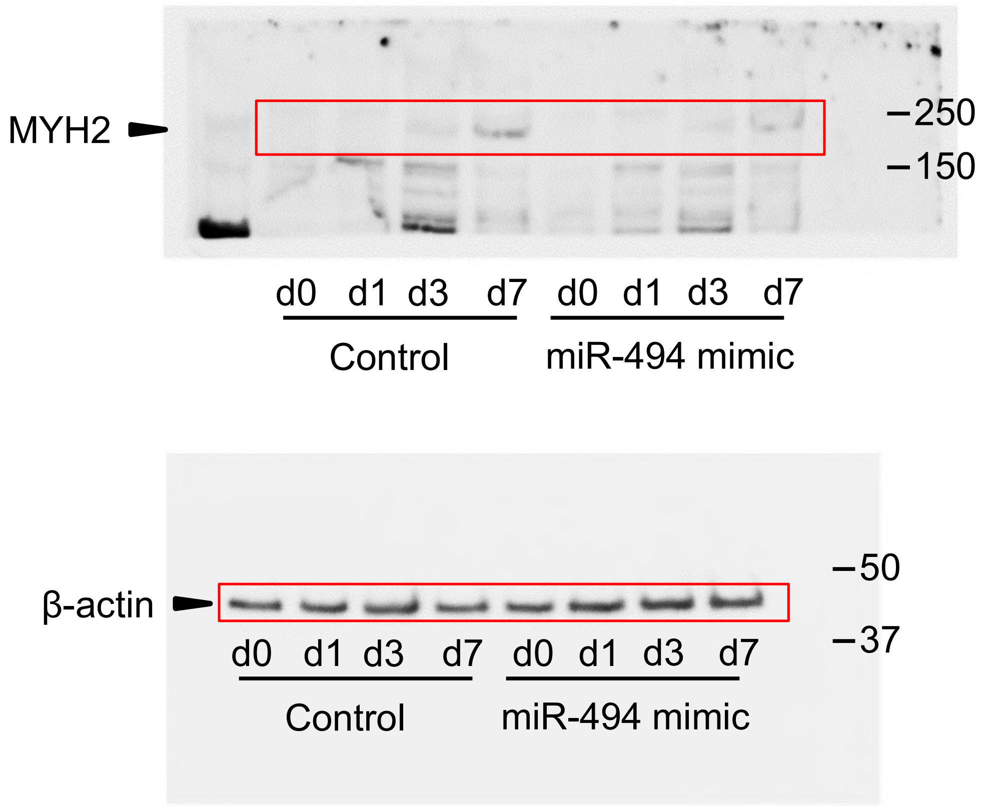


Original western blots for the images shown in Figure 1h. The regions of original blots used in main figures were encircled with red boxes.

## Supplementary Figure S7.

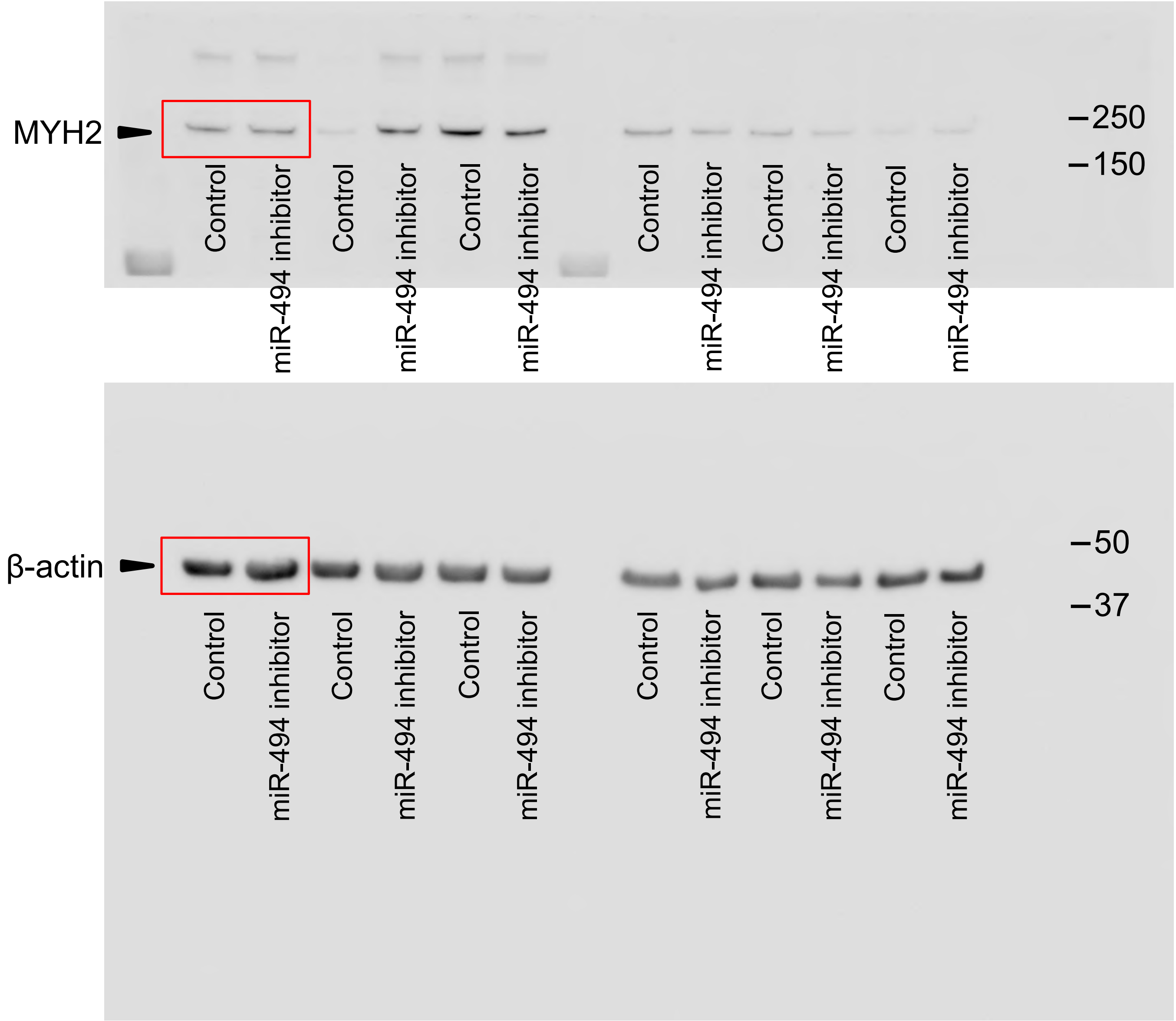


Original western blots for the images shown in Supplementary Figure 1b. The regions of original blots used in main figures were encircled with red boxes.

## Supplementary Figure S8 (to be continued).

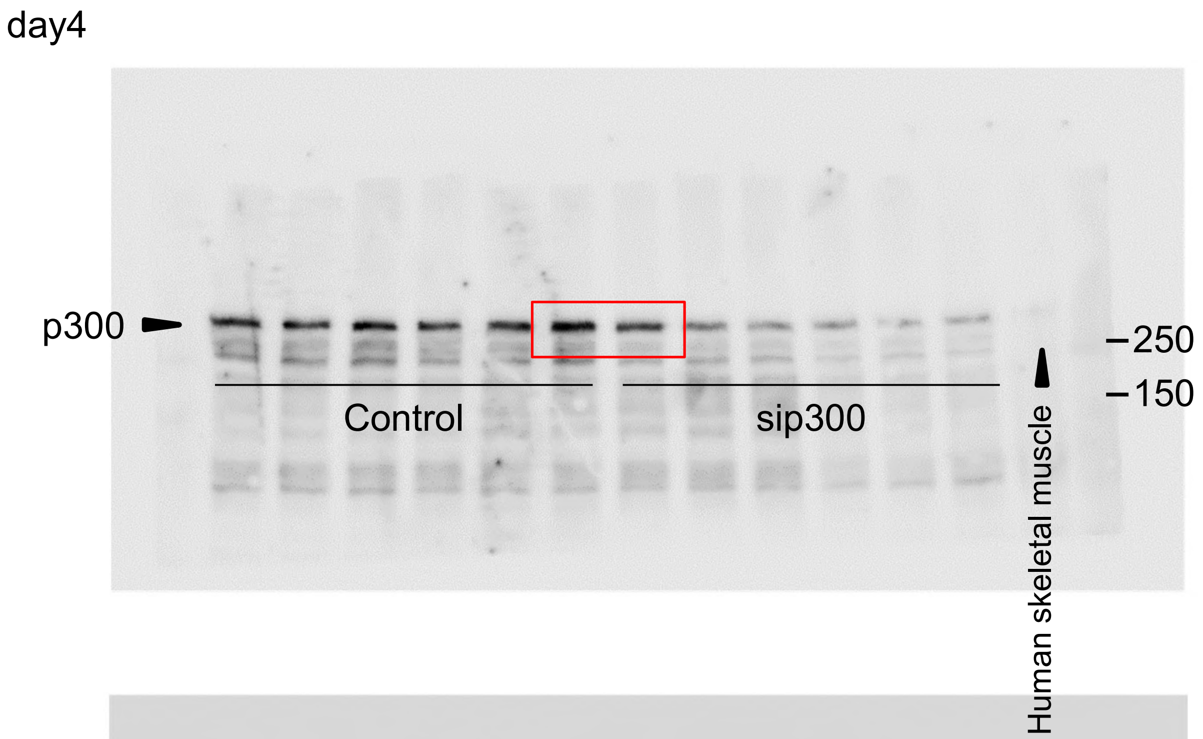


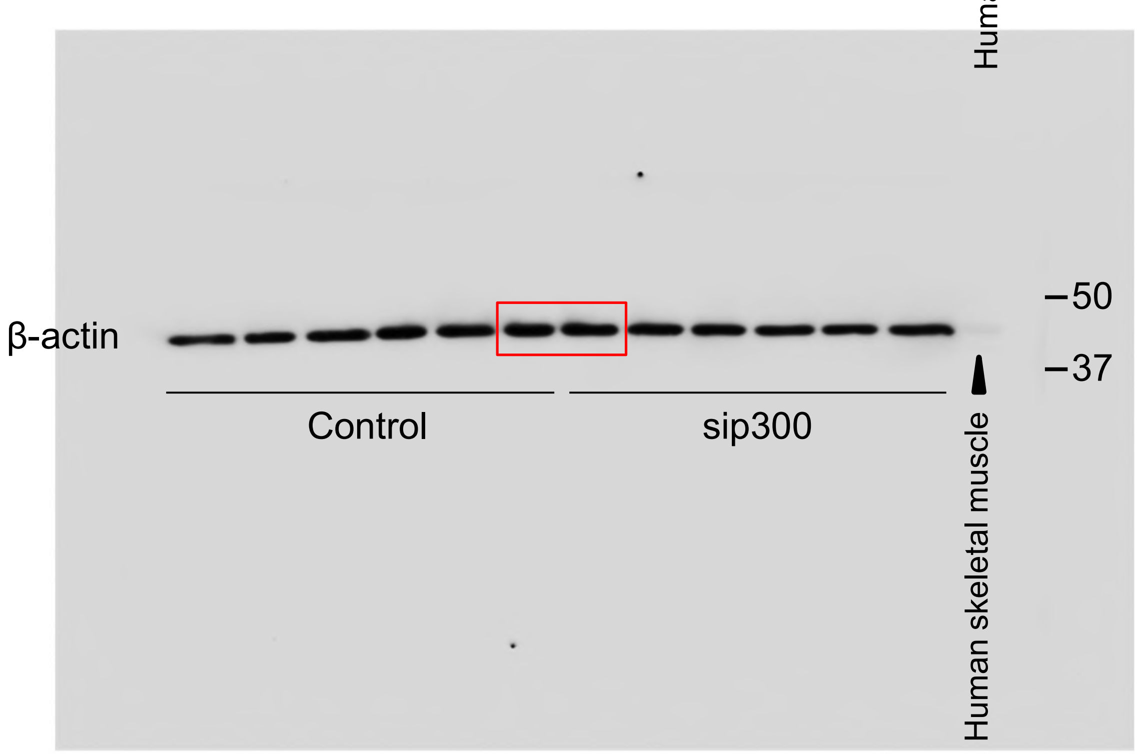


Original western blots for the images shown in Figure 2b. The regions of original blots used in main figures were encircled with red boxes.

## Supplementary Figure S8.

day7

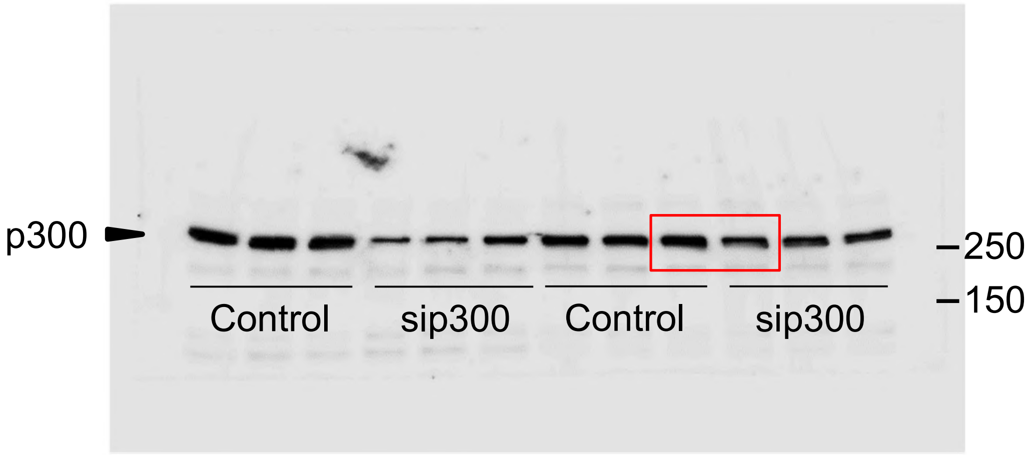


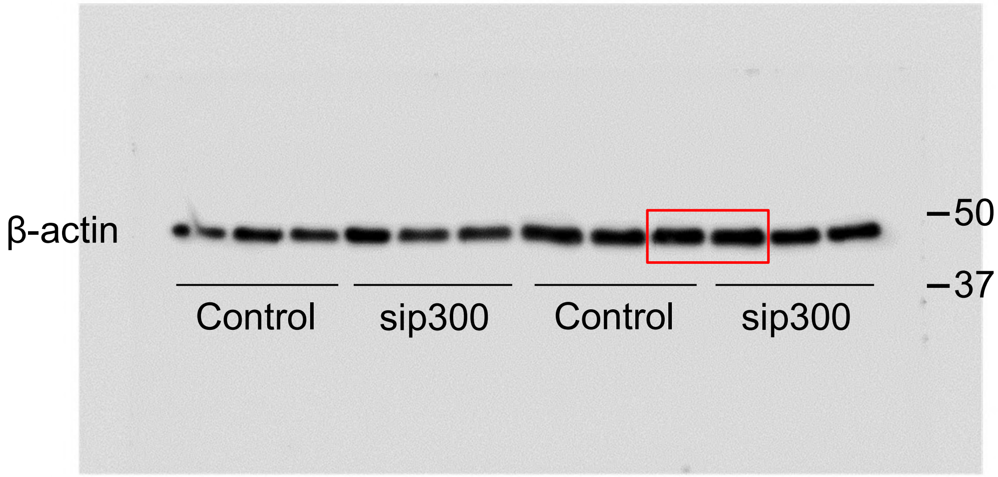


Original western blots for the images shown in Figure 2b. The regions of original blots used in main figures were encircled with red boxes.

## Supplementary Figure S9.

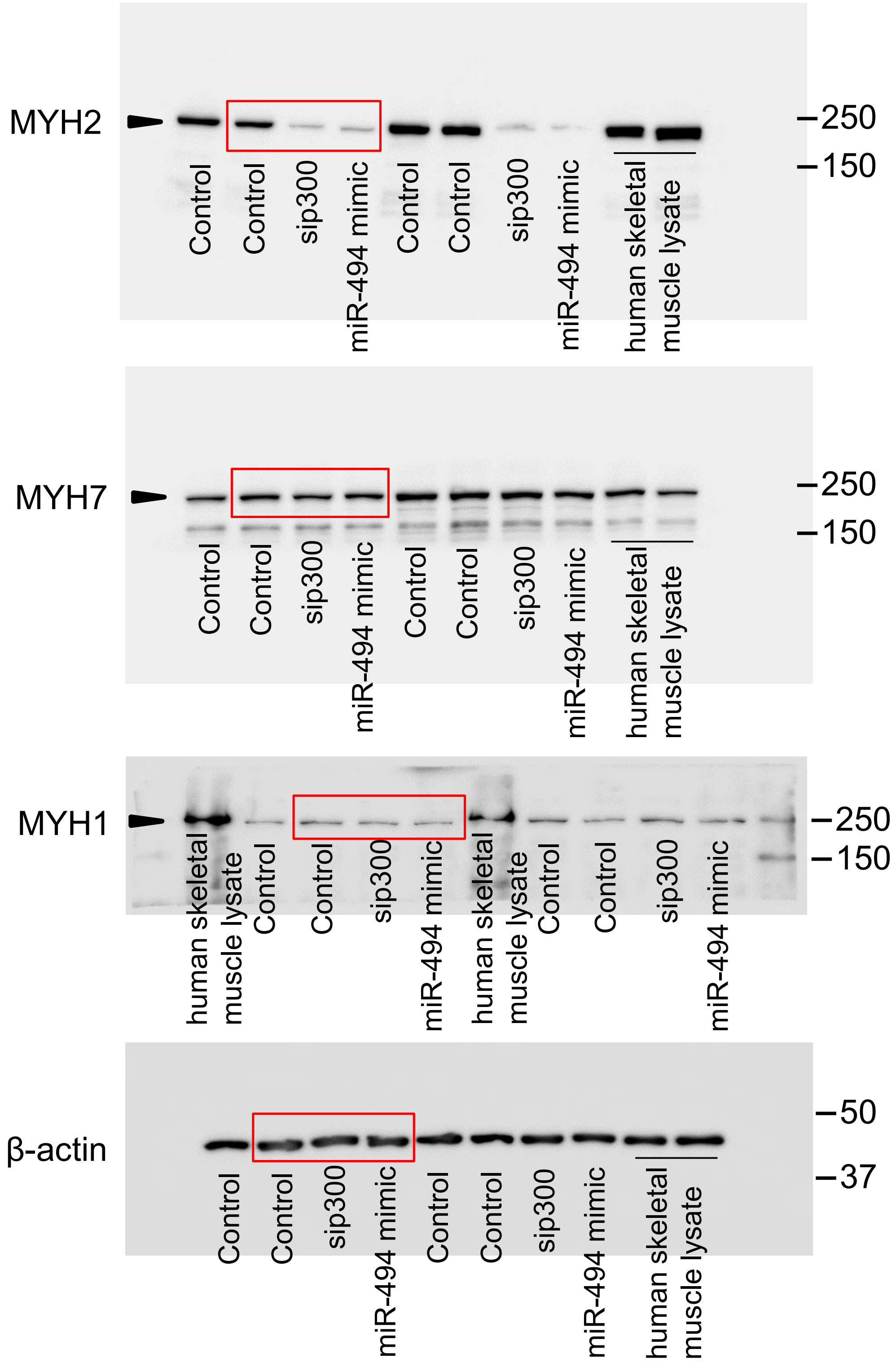


Original western blots for the images shown in Figure 2g. The regions of original blots used in main figures were encircled with red boxes.

## Supplementary Figure S10.

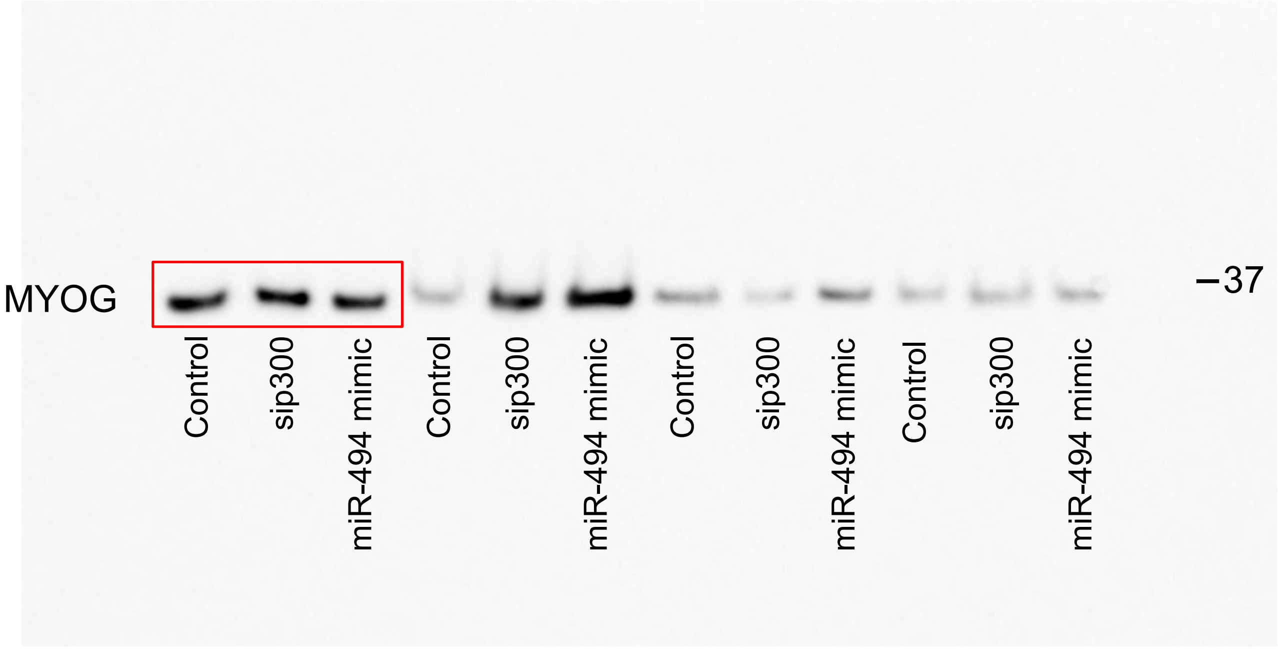


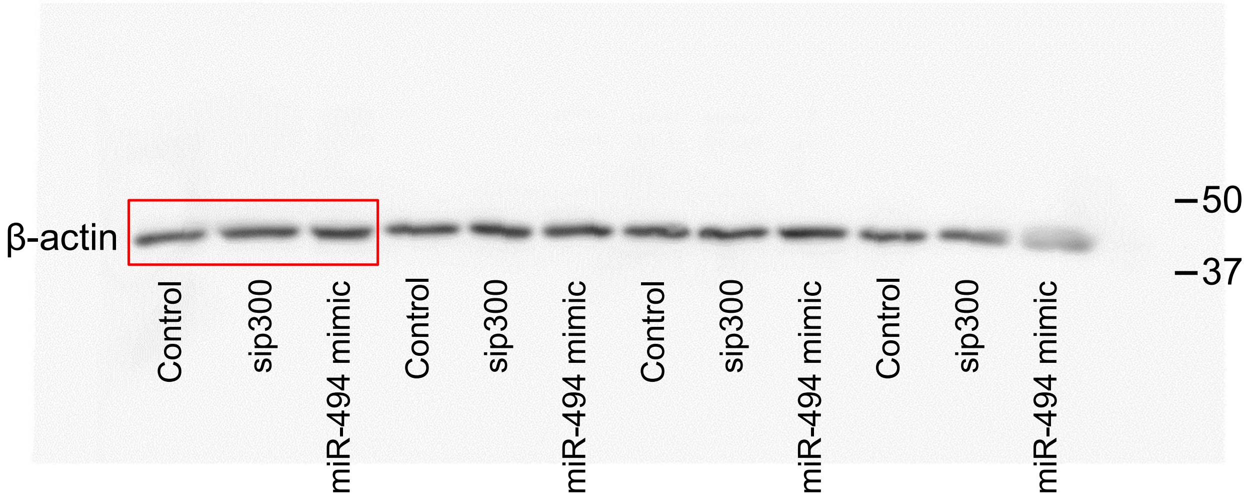


Original western blots for the images shown in Figure 2i. The regions of original blots used in main figures were encircled with red boxes.

## Supplementary Figure S11 (to be continued).

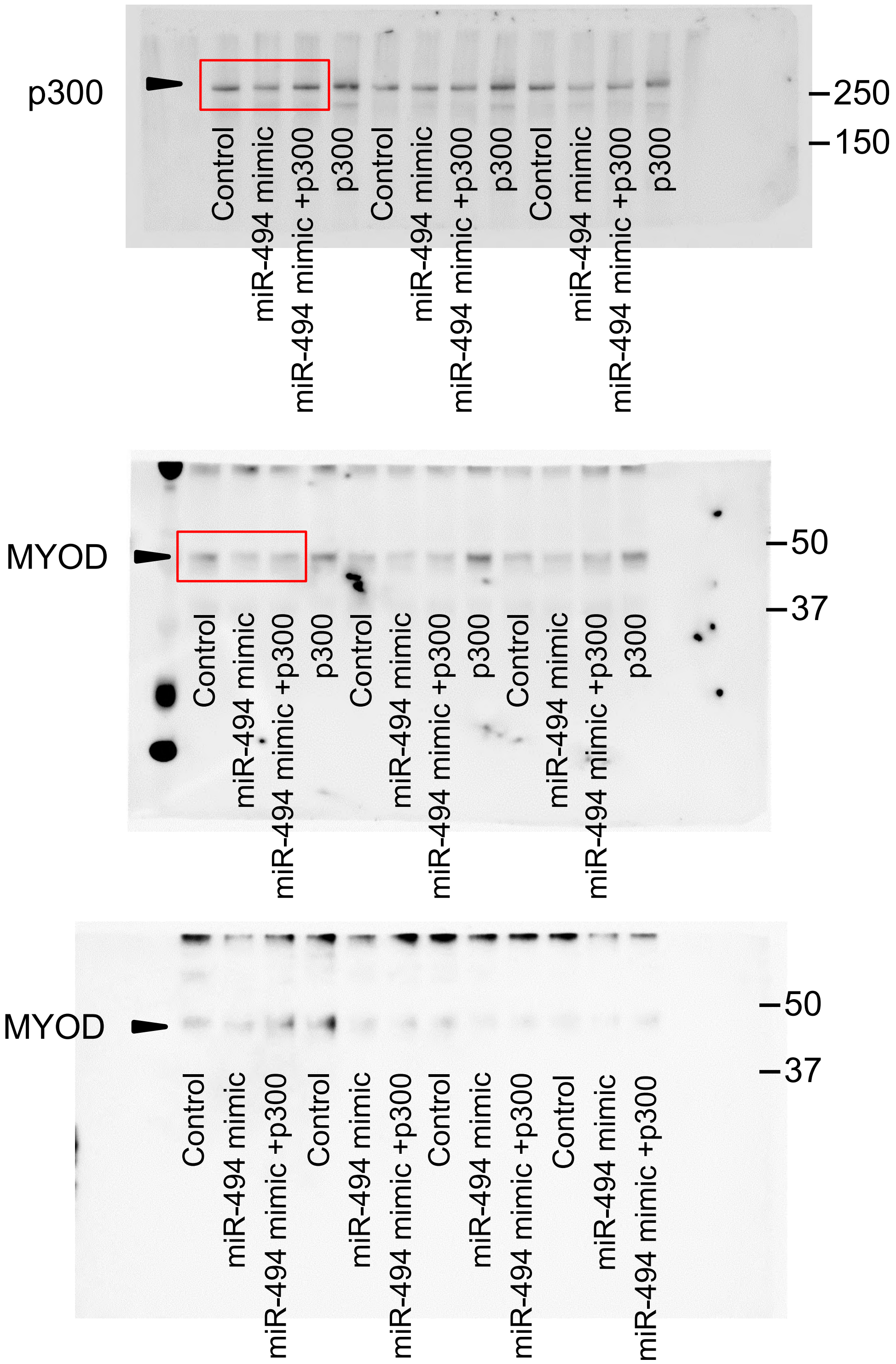


Original western blots for the images shown in Figure 4b. The regions of original blots used in main figures were encircled with red boxes.

## Supplementary Figure S11.

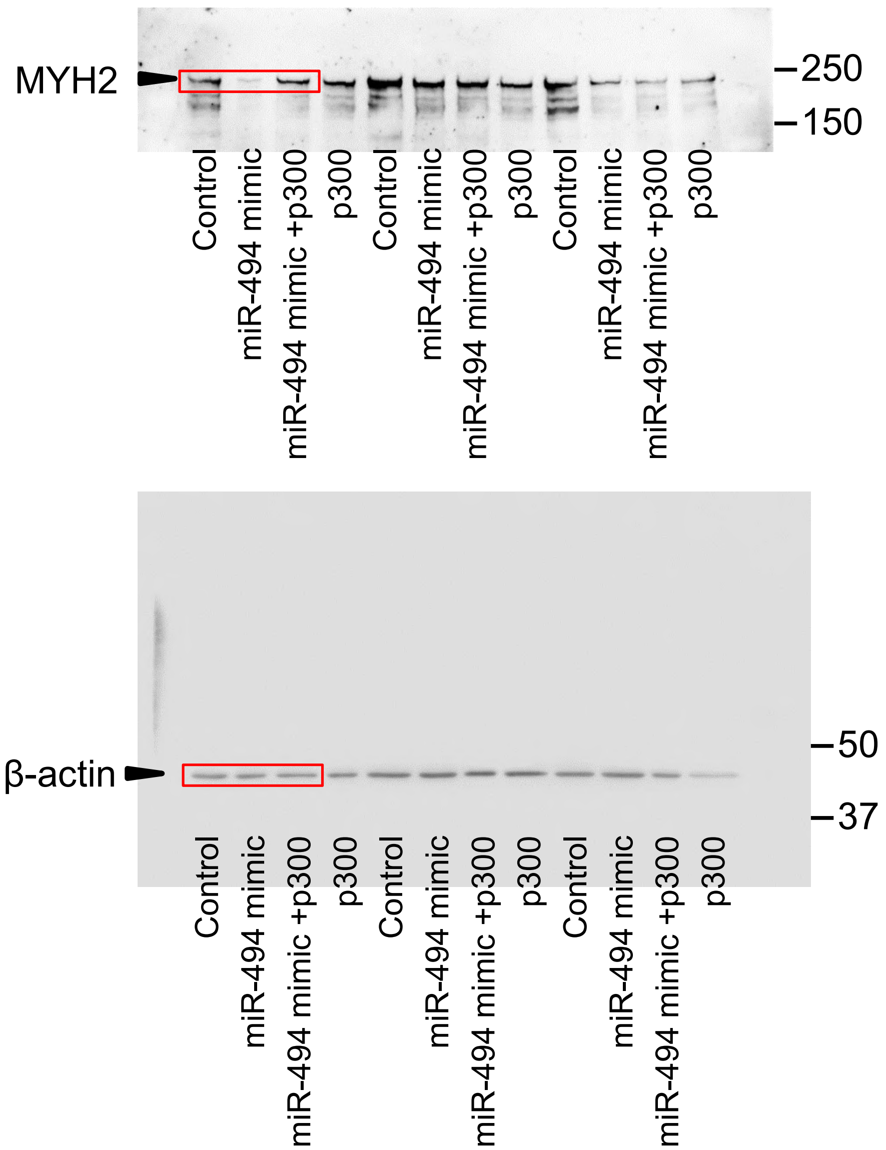


Original western blots for the images shown in Figure 4b. The regions of original blots used in main figures were encircled with red boxes.

## Supplementary Figure S12 (to be continued).

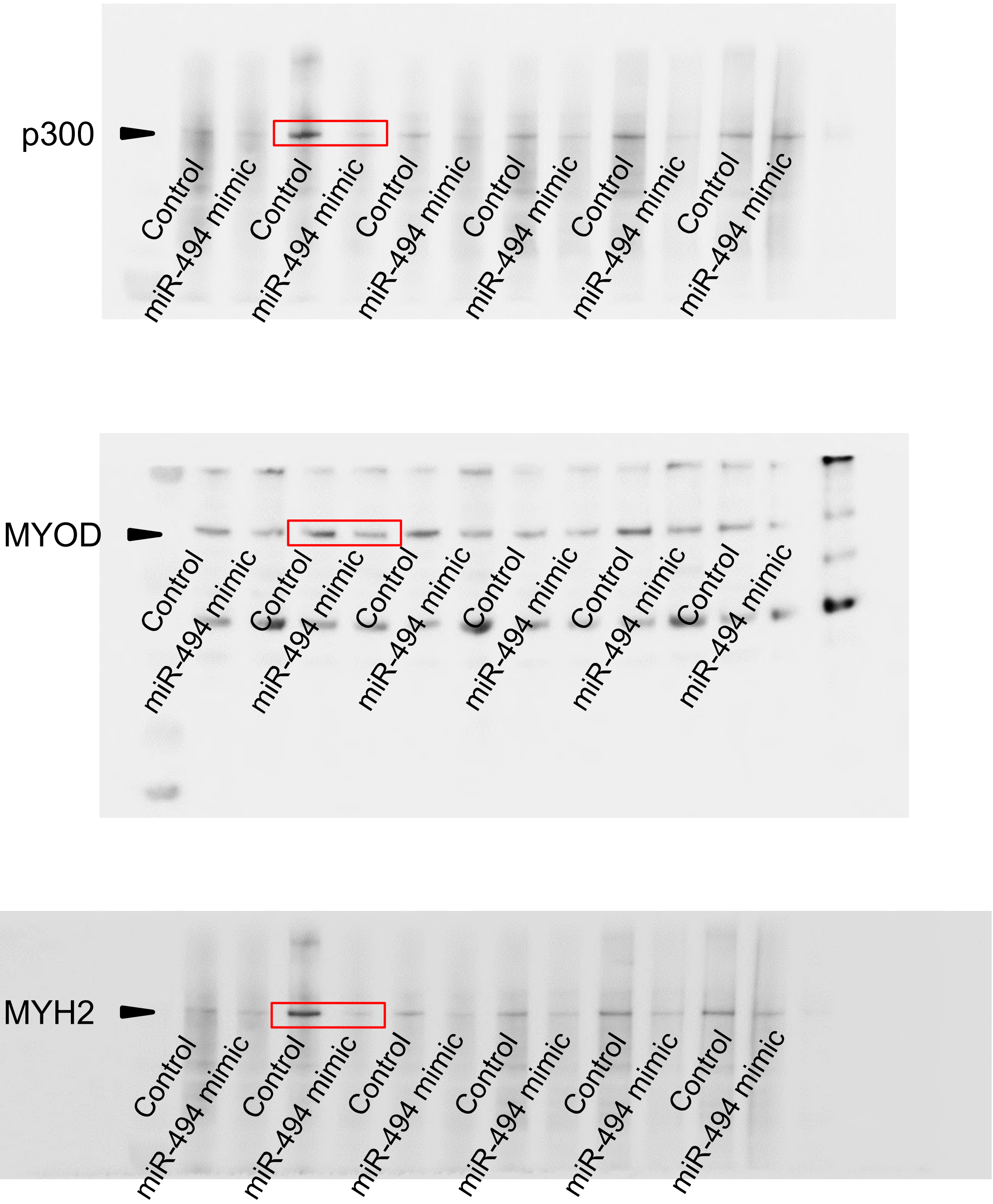


Original western blots for the images shown in Figure 6a. The regions of original blots used in main figures were encircled with red boxes.

## Supplementary Figure S12 (to be continued).

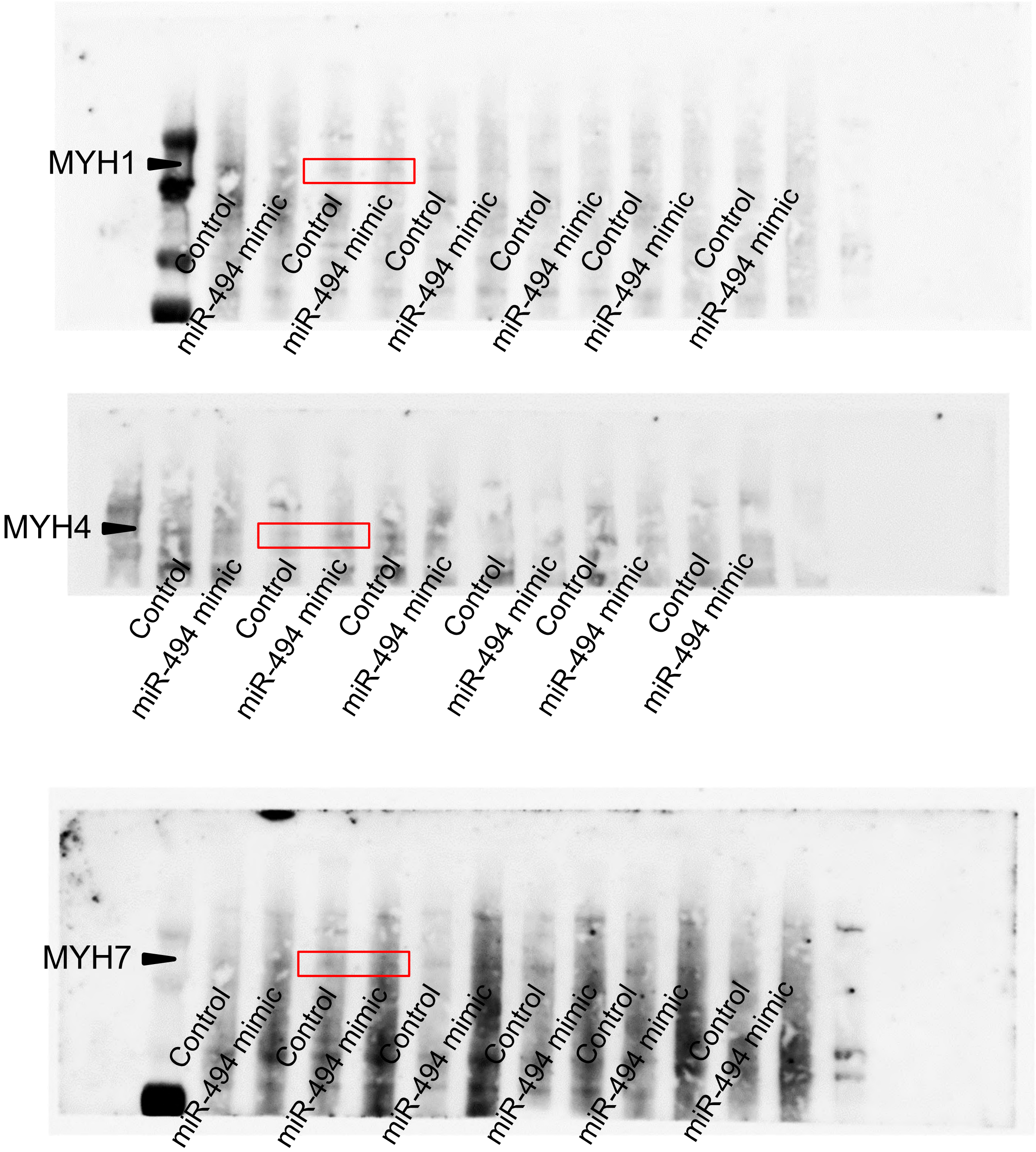


Original western blots for the images shown in Figure 6a. The regions of original blots used in main figures were encircled with red boxes.

## Supplementary Figure S12.

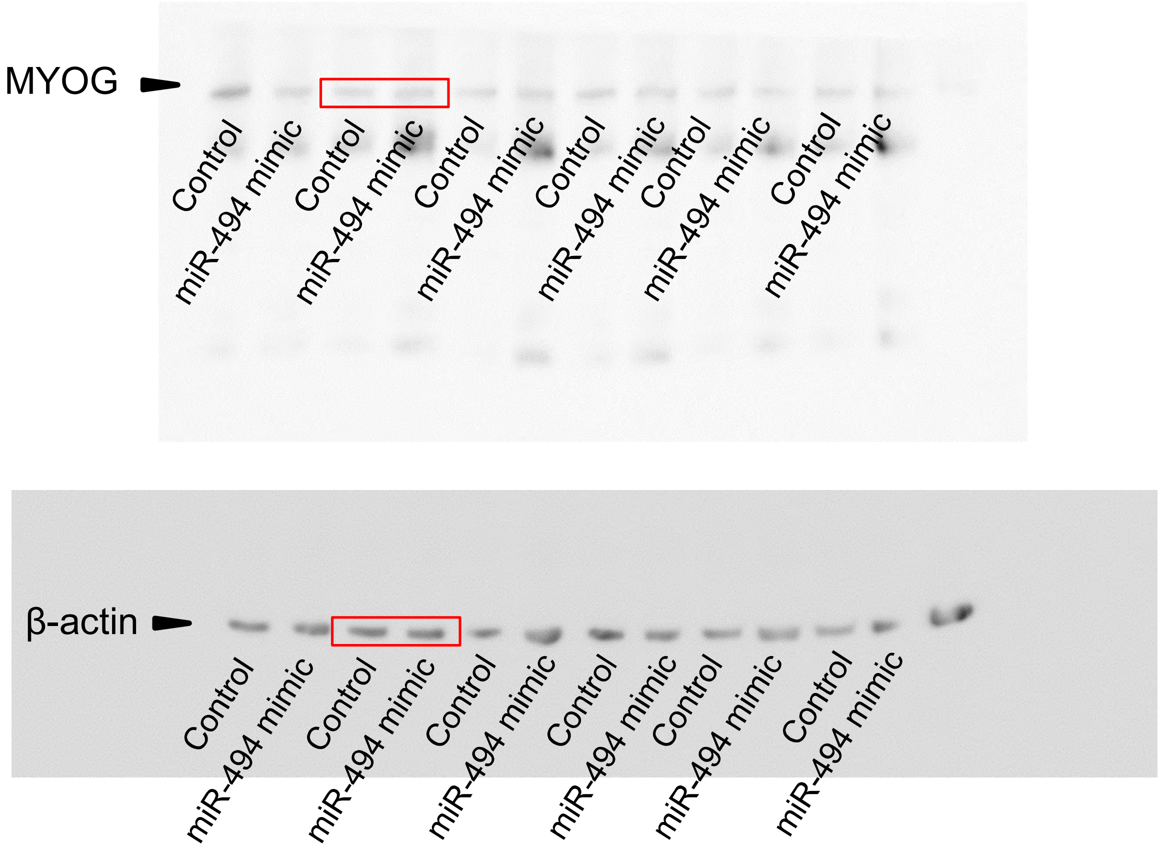


Original western blots for the images shown in Figure 6a. The regions of original blots used in main figures were encircled with red boxes.

## Supplementary Figure S13.

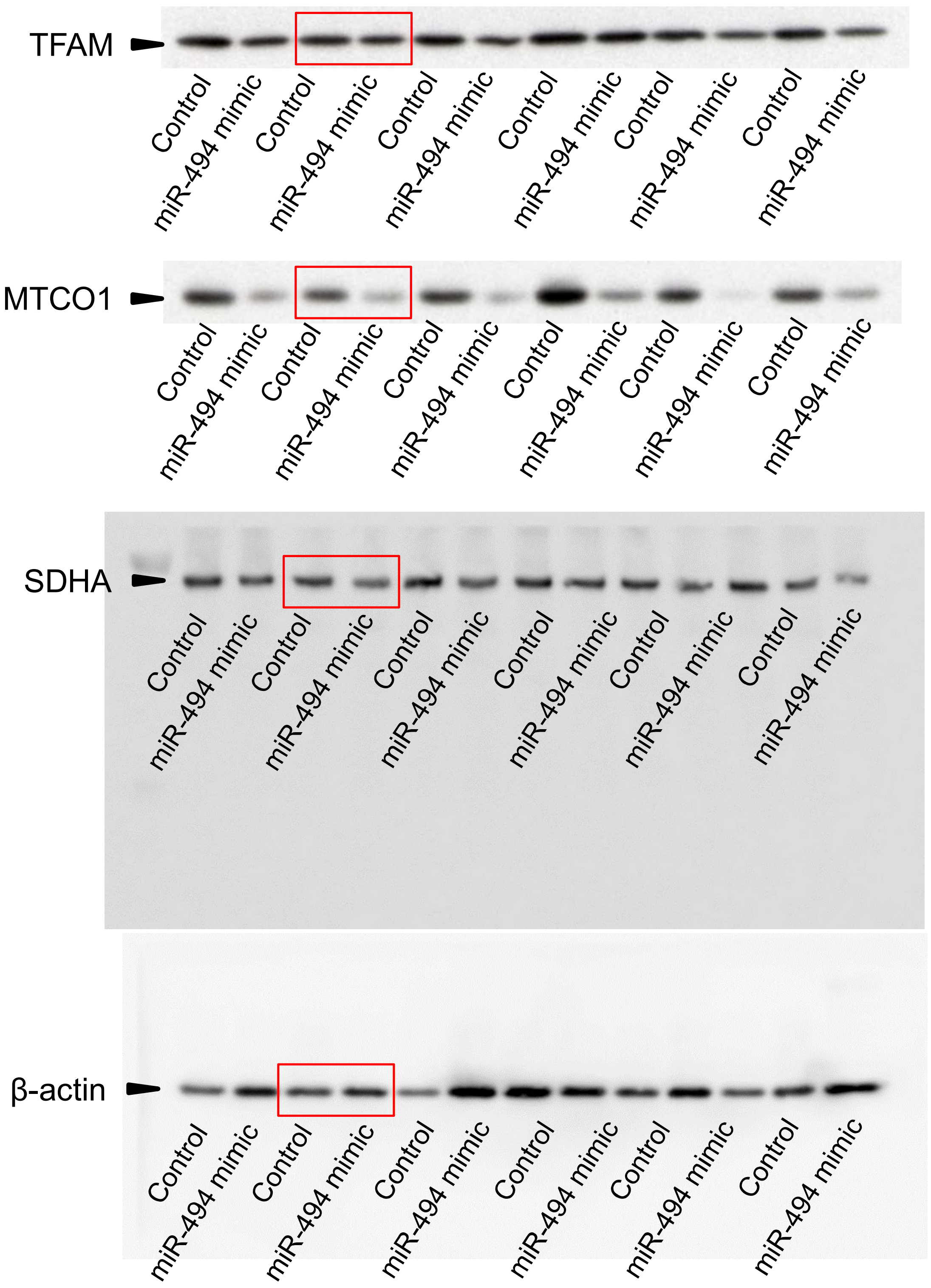


Original western blots for the images shown in Figure 6c. The regions of original blots used in main figures were encircled with red boxes.

# Supplementary Table S1.

| Gene | Forward (5' -> 3') | Reverse (5' -> 3') |
|---|---|---|
| *p300* | TTCAAACGCCGAGTCTTCTT | GTTGAGCTGCTGTTGGCATA |
| *β-actin* | CTCTTCCAGCCTTCCTTCC | CACCTTCACCGTTCCAGTTT |

**Supplementary Table S1.** Primers for qRT-PCR analysis
